# Anthropogenic modifications to fire regimes in the wider Serengeti‐Mara ecosystem

**DOI:** 10.1111/gcb.14711

**Published:** 2019-07-08

**Authors:** James R. Probert, Catherine L. Parr, Ricardo M. Holdo, T. Michael Anderson, Sally Archibald, Colin J. Courtney Mustaphi, Andrew P. Dobson, Jason E. Donaldson, Grant C. Hopcraft, Gareth P. Hempson, Thomas A. Morrison, Colin M. Beale

**Affiliations:** ^1^ Department of Earth, Ocean & Ecological Sciences University of Liverpool Liverpool UK; ^2^ Centre for African Ecology School of Animal, Plant and Environmental Sciences University of the Witwatersrand Johannesburg South Africa; ^3^ Department of Zoology & Entomology University of Pretoria Pretoria South Africa; ^4^ Odum School of Ecology University of Georgia Athens Georgia; ^5^ Department of Biology Wake Forest University Winston‐Salem North Carolina; ^6^ Natural Resources and the Environment, CSIR Pretoria South Africa; ^7^ Geoecology, Department of Environmental Sciences University of Basel Basel Switzerland; ^8^ Institutionen för arkeologi och antik historia Uppsala Universitet Uppsala Sweden; ^9^ York Institute for Tropical Ecosystems, Environment Department University of York York UK; ^10^ Ecology and Evolutionary Biology Princeton University Princeton New Jersey; ^11^ Institute of Biodiversity, Animal Health and Comparative Medicine University of Glasgow Glasgow UK; ^12^ South African Environmental Observation Network (SAEON), Ndlovu Node Phalaborwa South Africa; ^13^ Department of Biology University of York York UK

**Keywords:** conservation, fire regime, management, overgrazing, protected areas, savannah, Serengeti

## Abstract

Fire is a key driver in savannah systems and widely used as a land management tool. Intensifying human land uses are leading to rapid changes in the fire regimes, with consequences for ecosystem functioning and composition. We undertake a novel analysis describing spatial patterns in the fire regime of the Serengeti‐Mara ecosystem, document multidecadal temporal changes and investigate the factors underlying these patterns. We used MODIS active fire and burned area products from 2001 to 2014 to identify individual fires; summarizing four characteristics for each detected fire: size, ignition date, time since last fire and radiative power. Using satellite imagery, we estimated the rate of change in the density of livestock *bomas* as a proxy for livestock density. We used these metrics to model drivers of variation in the four fire characteristics, as well as total number of fires and total area burned. Fires in the Serengeti‐Mara show high spatial variability—with number of fires and ignition date mirroring mean annual precipitation. The short‐term effect of rainfall decreases fire size and intensity but cumulative rainfall over several years leads to increased standing grass biomass and fuel loads, and, therefore, in larger and hotter fires. Our study reveals dramatic changes over time, with a reduction in total number of fires and total area burned, to the point where some areas now experience virtually no fire. We suggest that increasing livestock numbers are driving this decline, presumably by inhibiting fire spread. These temporal patterns are part of a global decline in total area burned, especially in savannahs, and we caution that ecosystem functioning may have been compromised. Land managers and policy formulators need to factor in rapid fire regime modifications to achieve management objectives and maintain the ecological function of savannah ecosystems.

## Graphical abstract

Fire has declined globally, and our study documents these changes taking place within the boundaries of one of Africa's most important transboundary protected areas. We find striking changes to the fire regime over time, with the number of fires declining by 40%, and the area burnt by 39%, in 14 years. We attribute the decline in fire to human‐induced land use changes, particularly increases in cattle density in some areas of the system. These changes, and the underlying driver (livestock) are likely particularly widespread across Africa.

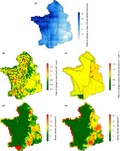

## 1 INTRODUCTION

Fire has been a natural ecological process for hundreds of millions of years (Bond, 2005; Bowman et al., 2009), interacts with human activities (Archibald, Staver, & Levin, 2012) and is a key ecological and evolutionary driver (Bond & Keeley, 2005). Fire influences the distribution of biomes (Bond, Woodward, & Midgley, 2005), carbon sequestration (Williams, Hutley, Cook, Russell‐Smith, & Chen, 2004), nutrient exchange (Frost & Robertson, 1987) and vegetation structure (Govender, Trollope, & Wilgen, 2006). Burning is formally and informally used as a management tool in many flammable ecosystems, both inside and outside protected areas (Parr, Robertson, Biggs, & Chown, 2004). Understanding the spatio‐temporal patterns exhibited by fire, the factors driving fire occurrence, and the extent to which fire characteristics can be manipulated is essential for the successful management of fire‐prone ecosystems, particularly given increasing human pressures and climate change (Bowman et al., 2009).

Savannah ecosystems cover approximately half of the African continent (Parr, Lehmann, Bond, Hoffmann, & Andersen, 2014). Fire is one of the most common (in some cases only) management tools used in African savannahs (Beale et al., 2018). Despite this, there is widespread debate concerning fire management, with approaches ranging from complete fire suppression (e.g. by the Kenya Wildife Service), burning to control woody encroachment (e.g. Gabon), and ‘burning for biodiversity’ (e.g. South Africa), where fire‐driven patchiness is used as a tool for maximizing biodiversity (Beale et al., 2018; Parr & Brockett, 1999). The overall effects of fire management at regional scales remain unknown. For example, one study of a 45 year interval in Kruger National Park, South Africa found that variation in the area burnt was dependent on rainfall and not management objectives, even though managers were able to influence the seasonality of fire (Smit, Smit, Govender, Linde, & MacFadyen, 2013; van Wilgen, Govender, Biggs, Ntsala, & Funda, 2004). The context‐dependent nature of fire, however, means that this finding may not be globally applicable. Many studies focus on a single variable to describe a fire regime, span a limited temporal range or do not include changes at regional scales and among several management (although see Buthelezi, Mutanga, Rouget, & Sibanda, 2016; Tarimo, Dick, Gobakken, & Totland, 2015). There is, therefore, a need for studies that document fire regimes and their drivers more widely and in specific regions of high socio‐economic importance (Archibald, Roy, Wilgen, & Scholes, 2009; Beale et al., 2018; van Wilgen et al., 2004).

Examining the multidimensionality of fire is crucial in understanding fire as a component of the ecology of an ecosystem. For example, individual fires can be characterized by their size, seasonality, return interval and intensity (Gill, 1975). The long‐term patterns in these characteristics describe the fire regime (Bond & Keeley, 2005; Hempson et al., 2018). Fire regimes vary by both broad‐ and fine‐scale environmental factors, including climate (Balfour & Howison, 2002), vegetation (Archibald et al., 2009), herbivory (Archibald, Nickless, Govender, Scholes, & Lehsten, 2010) and topography (Wood, Murphy, & Bowman, 2011). At large spatial scales fire regimes are driven by environmental factors, but at finer scales human activities also influence burning (Archibald, Lehmann, Gomez‐Dans, & Bradstock, 2013; Archibald, Nickless, et al., 2010; Archibald, Scholes, Roy, Roberts, & Boschetti, 2010; Smit et al., 2013). Humans increase the number of ignitions, and broaden the times of year when ignitions happen, but also inhibit fire spread by fragmenting landscapes and reducing fuel load through livestock grazing (Archibald, 2013; Archibald, Scholes, et al., 2010; Frost, 1999; Guyette, Muzika, & Dey, 2002). Diverse socio‐economic, cultural, political and environmental conditions result in great variability in the motives behind anthropogenic burning, in the practice of how burns are applied, and on the consequences for fire regimes (Bowman et al., 2011; Laris, 2002; Le Page, Oom, Silva, Jönsson, & Pereira, 2010). Determining how people influence fire regimes is especially important given increasing human population pressures and associated land‐use changes in savannahs (Archibald, Scholes, et al., 2010).

Covering nearly 33,000 km^2^, the Serengeti‐Mara ecosystem of southern Kenya and northern Tanzania is one of the largest transboundary protected areas in the world. This savannah system burns frequently and the importance of fire for the ecology of the ecosystem is well documented (e.g. Dublin, 1995; Holdo, Holt, & Fryxell, 2009). The ecosystem is characterized by contrasting spatial gradients in rainfall and soil nutrients and comprises multiple management units with different fire management approaches. Consequently, there is great spatial variability in the drivers of fire across the ecosystem. Substantial historical changes to the fire regime of the Serengeti‐Mara have been attributed to the recovery of the wildebeest (*Connochaetes taurinus* Burchell, 1823) population from rinderpest (Sinclair et al., 2007). During the mid‐19th century, the rapidly increasing wildebeest population consumed large quantities of grass biomass, leading to reductions in the total area burned each year, enhancing tree recruitment and increasing woody cover (Dublin, 1995). Whilst the wildebeest population stabilized at 1.3 million animals during the past decades (Hopcraft et al., 2015), burgeoning human populations surrounding the ecosystem's protected areas continue to alter land‐use patterns (Estes, Kuemmerle, Kushnir, Radeloff, & Shugart, 2012), rainfall has increased across the broader region (Ogutu, Bhola, Piepho, & Reid, 2006), and there have been changes in the management of some protected areas (Sinclair et al., 2007). It is, therefore, likely that there have been recent changes in the Serengeti‐Mara's fire regime, the scale and causes of which are as yet undocumented.

We use satellite Earth Observation products to describe the fire regime across the broader Serengeti‐Mara ecosystem and investigate its spatio‐temporal drivers (see Dempewolf, Trigg, DeFries, & Eby, 2007). We examine how six characteristics of the Serengeti‐Mara's fire regime (fire size, ignition date, time since last fire, radiative power, total number of fires and total area burned) vary through space and time, both across the ecosystem and within its component management units, and investigate the drivers of these spatio‐temporal patterns. Specifically, our objectives are to: (a) characterize spatiotemporal variation in fire regimes across the wider Serengeti‐Mara ecosystem over a 14 year period (2001–2014), and (b) determine the biotic and abiotic factors driving these patterns. We predicted that the combination of strong environmental gradients and differences in management approaches will produce high variability in the observed patterns of fire across Serengeti‐Mara. We anticipated that rainfall would be the primary driver of these patterns and that human activities, particularly reductions in fuel loads by livestock grazing, would have a detectable influence on certain aspects of the fire regime.

## 2 METHODS

### 2.1 Study area

**Figure 1:**
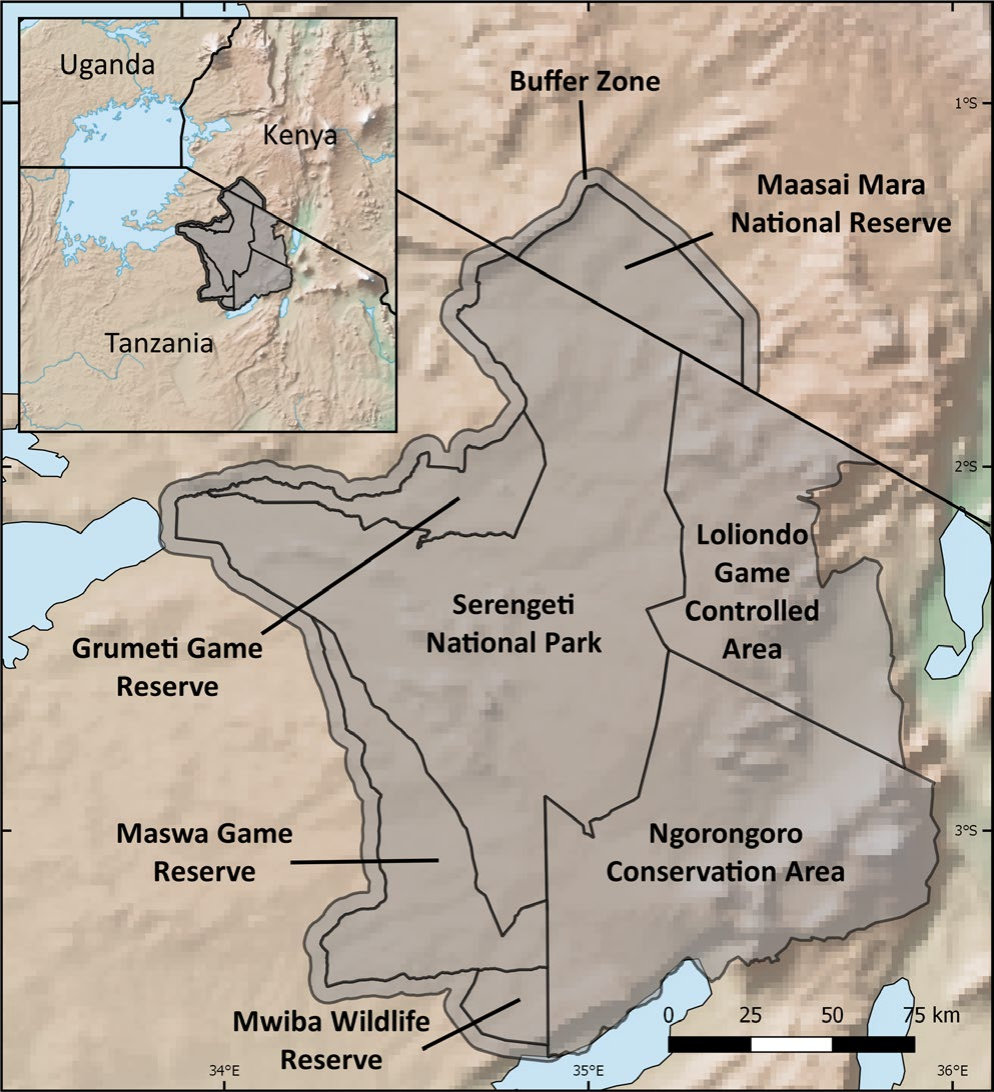
Map of the study area with the management units (shaded) labelled and inset a map of equatorial eastern Africa showing the location of the study area. Note the 5 km wide buffer except around Loliondo and Ngorongoro to the east. The base map is of elevation (made with Natural Earth)

We defined our study area (Figure 1) as the protected areas of the Serengeti‐Mara ecosystem and included a 5 km buffer around the Maasai Mara National Reserve, Serengeti National Park (SNP), Grumeti Game Reserve, Maswa Game Reserve and Mwiba Wildlife Reserve to allow us to compare protected areas to the de facto land management that takes place in the absence of formalised management institutions and agency. The buffer zone did not extend around Loliondo Game Controlled Area and Ngorongoro Conservation Area, as these protected areas contain significant settlements within their boundaries. The resulting region covers 36,305 km^2^, of which 91.5% (33,232 km^2^) is encompassed by protected areas. For the purposes of this analysis, each of the seven protected areas and the 5 km wide buffer zone were counted as a discrete management unit. Grumeti and Ikorongo Game Reserves were combined (hereafter ‘Grumeti Game Reserve’), because both are managed by the same organization. Fire management approaches differ between protected areas: the Maasai Mara follows a policy of active fire suppression; managers in Grumeti, SNP and Maswa actively burn (for a variety of reasons and with varying levels of control); whilst managers in NCA and Loliondo adopt a more localized approach, with most fires being lit by communal farmers.

### 2.2 Data sources

#### 2.2.1 Soil type

Soils are a proxy for nutrient availability and texture, playing an important role in determining vegetation structure and species composition, both of which affect fire (Anderson & Talbot, 1965). Soil data were downloaded from the FAO/UNESCO Digital Soil Map of the World (Fischer, Nachtergaele, Prieler, Velthuizen, & Verelst, 2008) and a soil type for each fire was extracted from the ‘dominant soil’ field.

#### 2.2.2 Elevation and slope

Topography either facilitates or hinders fire spread and affects characteristics such as radiative power (Pyne, Andrews, & Laven, 1996). We used the 30 m resolution NASA ASTER Global Digital Elevation Map (Meyer, 2011) to extract elevation and slope values for each fire.

#### 2.2.3 Primary productivity

Fuel load is one key factor determining the characteristics of fire and is the medium through which drivers such as rainfall, soil type and herbivory influence fire. Net primary productivity (NPP) is a useful measure of the local rate of accumulation of grass biomass (Running et al., 2004). Raster layers of annual NPP were downloaded from the Land Processes Distributed Active Archive Centre using Echo Reverb in the form of MODIS 1 km NPP MOD173 and rasters of monthly gross primary productivity were downloaded from the Numerical Terradynamic Simulation Group at the University of Montana (MOD17 A2) (Running & Zhao, 2015).

#### 2.2.4 Grass structure

We collected data on grass height from 15 50 × 50 m (0.25 ha) plots in Grumeti Game Reserve. We surveyed each plot six times between August 2016 and January 2017 and sampled grass height every 2 m along two 50 m line transects on each plot. For each point we computed the mean and rate of change in grass height. We aggregated to transect level using the median, and then used Google Earth Engine (Gorelick et al., 2017) to estimate grass height across the Serengeti‐Mara Ecosystem from Sentinel 1 Synthetic aperture radar data (Copernicus, 2018). We filtered Sentinel 1 data across Serengeti for ascending passes between 1 August 2016 and 30 June 2017, resulting in a total of 93 images, computing the 10th and 90th percentiles and the difference between them. We filtered locations where radar scatter in the lower percentile was over 23 to exclude bushes and trees. We fitted Classification and Regression Trees (with the Earth Engine CART algorithm) to predict grass height and grass growth rates across the Serengeti ecosystem using the computed 10th and 90th percentile of scatter, and difference between them (Gorelick et al., 2017). Correlations between modelled and predicted height were both >0.9, sufficient for an analysis across the ecosystem: the working Earth Engine script is available here.

#### 2.2.5 Wildebeest distributions

Grazing by the approximately 1.3 million wildebeest in the Serengeti‐Mara has a marked impact on grass structure, which in turn affects fire characteristics (Dublin, 1995; Holdo et al., 2009; Hopcraft et al., 2015). To include temporal distribution patterns of wildebeest, we aggregated a telemetry dataset of 54 GPS‐collared migratory wildebeest collected between 1999 and 2018 (see Hopcraft et al., 2014). To generate monthly population‐level wildebeest utilization maps, we first fit Brownian bridge movement models (BBMM) to trajectory data to generate utilization distributions (UD's) (Horne, Garton, Krone, & Lewis, 2007; Sawyer, Kauffman, Nielson, & Horne, 2009). BBMMs assume that movement trajectories are connected by Brownian motion between sequential, time‐specific GPS locations. Higher velocity movements result in more narrow distribution paths between points. Individual Brownian bridges for each individual were rasterized at a resolution of 500 m^2^ (median daily displacement of female wildebeest was 4.5 km) (Hopcraft et al., 2014). Because sample sizes varied across months in terms of number of data points and number of individuals, we reweighted raster data so that each month was represented equally. We did this in two steps: (a) dividing individual UD's into monthly Voronoi fractures (subdivisions of a plane based on the distance between points), based on the individuals’ GPS trajectory, and (b) inversely weighting each fracture by the minimum monthly number of GPS locations for that month, so that months with many GPS locations (across all individuals) had less weight than months with few GPS points. Next, we combined (i.e. summed) all individual UD's to generate monthly population‐level UD's and rescaled these surfaces so that total utilization summed to 1.0. Following estimation of population‐level UD's, we excluded cells containing the lower 5% of utilization values to remove areas with low probability of use (Sawyer et al., 2009).

#### 2.2.6 Livestock density

**Figure 2:**
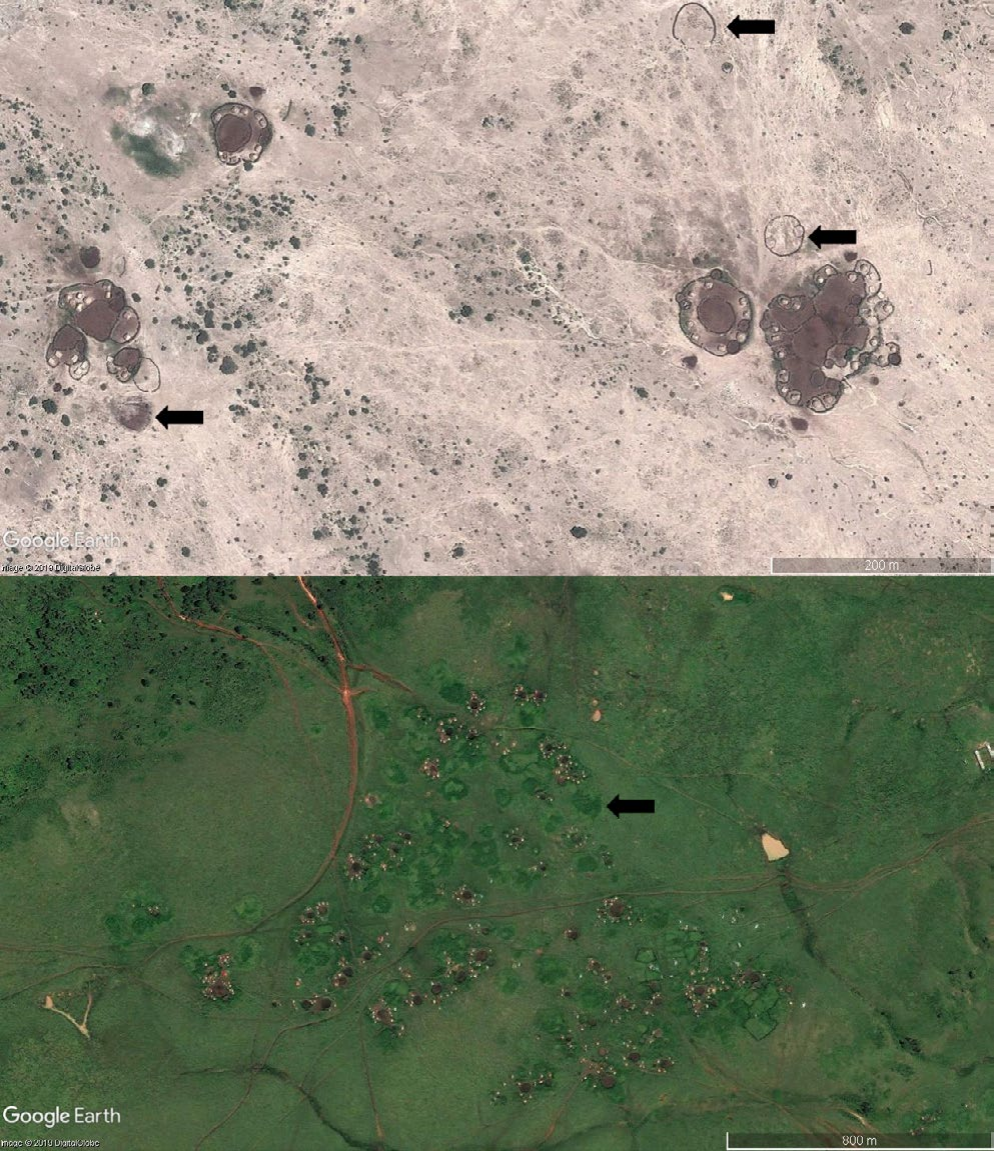
The differences between active *bomas* and scars (shown by arrows) in an area of low rainfall (top) and high rainfall (bottom). Note the multiple cells in *bomas* in the top image

It is illegal to graze livestock within Game Reserves and National Parks in Tanzania, but in practice this is difficult to enforce and encroachment along borders persists. Reliable and extensive data on livestock distribution across our study area were not available and we, therefore, established the density of active *bomas* as a proxy for livestock density. *Bomas* are livestock enclosures, generally constructed of thorny scrub. The ‘scar’ left by a *boma* persists for decades after the *boma* has been abandoned (Veblen, 2013), for this reason we defined an active *boma* using two visual criteria: (a) a clear contrast between the colour of the substrate within the *boma* and the colour of the substrate surrounding it (livestock trampling disturbs substrate and changes its appearance), and (b) a continuous fenced perimeter delimiting the *boma*. Each *boma* may comprise multiple internal ‘cells’, either to separate cattle (*Bos Taurus* Linnaeus, 1758), goats (*Capra aegagrus hircus* Linnaeus, 1758) and sheep (*Ovis aries* Linnaeus, 1758), or to accommodate the livestock of an extended group of people. Where this was the case, we counted the structure as a single *boma* rather than counting each cell individually (Figure 2). We used Google Earth (2017) to identify areas where two or more satellite images from different years overlapped. By visually counting the number of active *bomas* in each satellite image in the area of overlap, we could estimate the change in *boma* density through time and predict *boma* density across our study area and study period.

We processed the raw *boma* count data to generate a raster of the rate of change in *boma* density. We fitted a generalized linear model (GLM) to the raw count data using individual areas of overlap as the unit of analysis to estimate the rate of change in *boma* density over time. As we had an a priori assumption that management would affect the rate of change in *boma* density, we interpolated the rate of change in *boma* density from within protected areas and from the buffer zone separately. We used regression kriging, a method of weighting values by distance, with management unit as an auxiliary variable, to predict the rate of change in *boma* density within protected areas, and universal kriging to interpolate the rate of change in *boma* density within the buffer zone (Cressie, 1993). Our final products were a raster of the rate of change in *boma* density and a raster of the predicted *boma* density for each year of our study, both at 6 × 6 km resolution. We verified the results of our model by randomly selecting 25% (41) of the pixels for which our model had no observations, that is, pixels which were not covered by two or more overlapping satellite images. Where these pixels were covered by a single satellite image we counted the number of *bomas* within each pixel, if the pixel was not covered by a satellite image we randomly selected a different ‘no observation’ pixel. We then compared the *boma* density predicted by our model with the actual *boma* density in each ‘no observation’ pixel.

#### 2.2.7 Rainfall

We downloaded the 0.05° monthly rainfall product from the Climate Hazards Group InfraRed Precipitation with Station data (Funk et al., 2015) for our study interval (January 2001–December 2014). The effect of rainfall on fire differs depending on the temporal scale considered. The immediate effect of rainfall increases atmospheric and vegetative moisture resulting in smaller and cooler fires, whilst the longer term effect of rainfall increases grass biomass (fuel) and thereby increases fire size and intensity. We extracted values for rainfall during the month of the fire (monthly rainfall) and accumulated rainfall from the two rainfall years prior to the date of the fire (cumulative rainfall) (van Wilgen et al., 2004). These were used as covariates in our spatial models. We also created rasters of annual rainfall and used a GLM to estimate the rate of change in annual rainfall in each pixel. These were used as covariates in our temporal model.

#### 2.2.8 Fire data

The MODIS (moderate resolution imaging spectroradiometer) Active Fire (MCD14ML) and Burned Area products (MCD45A1) were obtained from the Land Processes Distributed Active Archive Centre at 500 m resolution for the period January 2001–December 2014. We combined the products to create a dataset of individual fires, their locations and associated fire characteristics. The dataset is described in detail in Hempson et al. (2018), but in summary: individual fires were identified using a flood‐fill algorithm (Archibald et al., 2009), with any spatially contiguous pixels that burned within 5 days of each other treated as a single fire. We calculated the centroid of each fire to represent it as a single spatial point, then we appended associated fire characteristics calculated from both MODIS products: fire size, ignition date (season), mean time since last fire (mean frequency) and radiative power (intensity).

Fire size was calculated as the number of pixels covered by a fire in the Burned Area Product. The date of ignition was calculated as the earliest date within a fire and was split into the calendar year (2001–2014), the ‘rainfall year’, and a value from 0 to 366 where 0 = 1st January and 366 = 31st December. A rainfall year ran from October (the start of the short rainy season) to September and is more ecologically meaningful than a calendar year, as it contains a full seasonal cycle. The time since the last fire at a given location was calculated by taking the mean value for all the pixels that had burned before a given fire. Because a pixel had to burn twice before a ‘time since last fire’ value could be calculated, fires early in the dataset are more likely to lack a value for the time since last fire. This adds an element of temporal bias and an expectation that time since last fire increases as a function of time. Also, time since last fire is maximally constrained by the duration of our dataset. Finally, the maximum radiative power (MW/km^2^) for any pixel within a fire was taken as a measure of fire intensity. Not all fires had an associated fire radiative power value due to differences in the detection probabilities of active fires versus burn scars (Krawchuk & Moritz, 2014). Fire radiative power data are only available if a fire was burning at the time of the satellite overpass and burn scars are not always detected if they are small (<250 × 250 m) or underneath tree canopies. Small fires are least likely to have a value for fire radiative power, which biases our dataset towards larger and, therefore, potentially hotter fires. Although missing data in both fire radiative power and fire return interval characteristics add known biases, they do not add spatial bias, only noise.

### 2.3 Analysis

#### 2.3.1 Spatial drivers of fire

To examine the spatial drivers of fire we used a Bayesian conditional autoregressive modelling approach using an integrated nested Laplace approximation (INLA). This approach allows us to explicitly account for spatial autocorrelation of predictor variables. INLA provides a computationally efficient framework for approximating posterior parameter estimates (Lindgren, Rue, & Lindström, 2011; Rue, Martino, & Chopin, 2009), while conditional autoregressive models have been found to perform well compared to spatial regression models (Beale, Lennon, Yearsley, Brewer, & Elston, 2010). We fitted a stochastic partial differential equation model for each fire characteristic, explaining the characteristic as a function of rainfall and predicted *boma* density, wildebeest utilization, slope, elevation, management unit, soil type, monthly GPP and annual NPP. To account for correlations between fire characteristics, we included them as covariates in our models. We split rainfall into two separate covariates: rainfall during the month of the fire (monthly rainfall) and cumulative rainfall from the beginning of the previous rainfall year to the date of the fire. We included all covariates as linear effects, except cumulative rainfall, which had both linear and quadratic terms, as it was unclear where the Serengeti‐Mara fell on the intermediate fire‐aridity curve (Pausas & Bradstock, 2007). To account for the effect of grazing on grass structure in the months preceding a fire, we included wildebeest distribution as a cumulative total for the month of the fire and the 2 months prior to this. We centred and scaled covariates and used vague priors for all model parameters. We used 95% credible intervals to assess support for the effect of each covariate. All analyses were run in r version 3.2.3 (R Core Team, 2012) using the r‐inla package (Martins, Simpson, Lindgren, & Rue, 2013).

#### 2.3.2 Temporal drivers of fire

We assessed temporal trends in four individual fire characteristics (size, ignition date, time since last fire and radiative power), and two aggregate fire characteristics (total number of fires and total area burned). We used Pearson's product‐moment correlations to assess annual trends across the whole ecosystem and conducted a spatially explicit analysis that calculated the rate of change in each fire characteristic across 6 × 6 km pixels (*n* = 1,152). To calculate rates of change we aggregated the characteristics for all the fires in each year into separate rasters and used GLMs to estimate the rate of change in each pixel over the period 2001–2014, and to predict a baseline area burnt for each pixel in 2001.

We fitted a GLM to predict the change in total area burned, using the rate of change in *boma* density, management unit, the mean and rate of change of annual rainfall and the baseline area burned in each pixel as predictors. Baseline area burned determines the capacity for the burned area of a pixel to change, whilst mean annual rainfall determines the resilience of a pixel to any changes in the mean annual rainfall or *boma* density. We anticipated that including interactions between these covariates would improve our model. From a full model containing all predictors we fitted a reduced model using single‐term deletions and log likelihood ratio tests. We performed a post hoc Tukey's honestly significant difference test to compare levels within our management covariate, used diagnostic plots to assess whether the residuals met the assumptions of all GLMs and tested our Poisson GLM for overdispersion. A table of the error structures, link functions and data transformations used for all GLMs can be found in Table S1. Finally, we used Pearson's product‐moment correlations to: (1) differentiate the influence of two significant covariates on the area burnt: (a) reducing the number of fires, or (b) reduction in fire size, and (2) to test our assumptions that higher predicted boma density decreased grass height but not grass growth rates.

## 3 RESULTS

### 3.1 Spatial patterns

**Figure 3:**
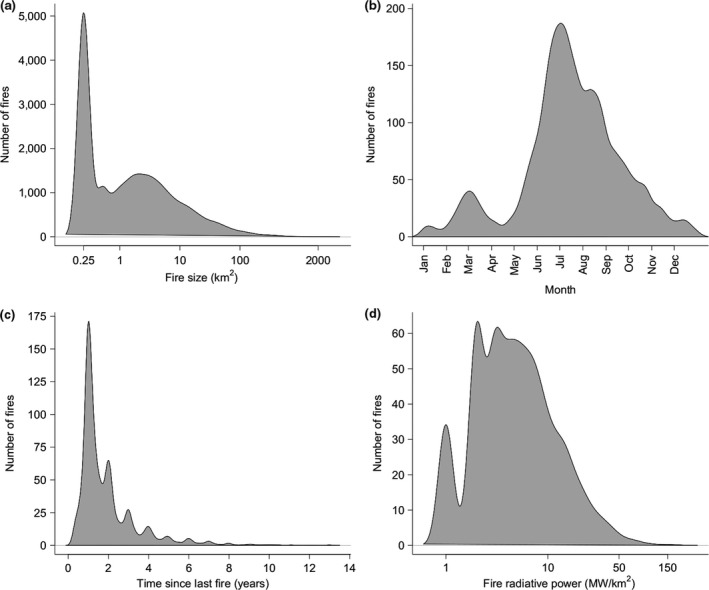
The distribution of fire traits: (a) fire size, (b) ignition date, (c) time since last fire and (d) fire radiative power

We detected 13,635 fires across our study site between 2001 and 2014. The median area burnt annually was 8,211.1 km^2^ (22.6% of the total study area) but differed considerably between protected areas and the buffer zone. In the buffer zone the median area burnt was 116 km^2^ (3.2%), but within protected areas it was 8,001 km^2^ (24.1%). Most fires were small: 37.2% occupied only 0.25 km^2^ (a single 500 × 500 m pixel), 78.4% were less than 5 km^2^ (20 pixels), whilst the largest (in SNP) occupied 2,316 km^2^ (9,263 pixels) (Figure 3a). Fire occurrence throughout the year was bimodal, with fire seasons matching the two dry seasons (Figure 3b; Figure S1). Time since fire varied widely, with a maximum of 13.1 years and a minimum 0.25 years. Over a quarter of the area, 10,383 km^2^ (28.6%) remained unburnt for the duration of the study, meaning the maximum time since last fire exceeds the 14 year duration of the study. Overall, 21.7%, 57.6% and 70.8% of fires occurred within 1, 2 and 3 years of the preceding previous fire, respectively, although some areas burnt twice within a 3‐month interval (Figure 3c). Due to limitations in the MODIS active fire product (see Methods) 2,869 fires (21%) had an associated value for radiative power. Of these, 2,847 fires (99.2%) had a radiative power of less than 50 MW/km^2^ (Figure 3d), which is comparable to the intensities of fires across southern Africa reported by Archibald, Nickless, et al. (2010).

**Figure 4:**
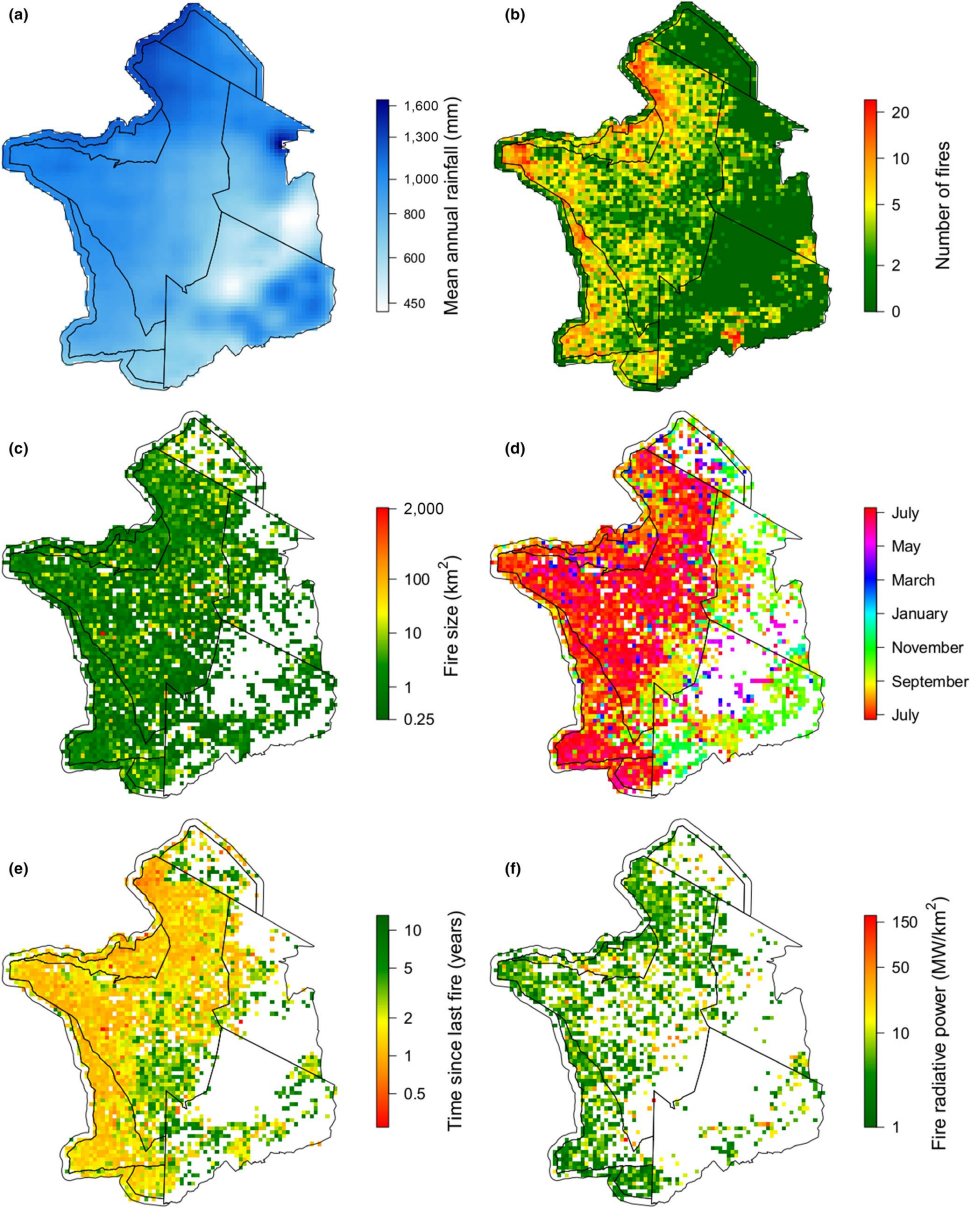
Spatial patterns in: (a) mean annual rainfall, (b) number of fires; note fires are much rarer in the Maasai Mara, (c) median fire size, (d) median ignition date, note the east–west pattern, (e) median time since last fire; note the short grass plain in south‐eastern SNP and the difference between Maasai Mara and northern SNP and (f) median fire radiative power

There was an east–west gradient in fire occurrence that correlated with the spatial pattern in mean annual rainfall (Figure 4a). The number of fires was particularly high in the west and north–west of SNP, with few fires occurring in the Maasai Mara, Loliondo Game Reserve and Ngorongoro Conservation Area, (although Ngorongoro Crater is visually distinguishable) (Figure 4b). There was also an east–west pattern in the seasonality of fires, with fires across Mwiba, Maswa, SNP and Grumeti concentrated in the long dry season (June–August) and fires in the NCA, Loliondo and to some extent Maasai Mara occurring at the start of the short rains (September–December) (Figure 4d). Large fires (>50 km^2^) were most common in the Maasai Mara, but also occurred throughout the ecosystem (Figure 4c). Maasai Mara and NCA had longer times since last fire than the other regions, although the short‐grass plains are clearly distinct from the surrounding landscape (Figure 4e). Fires were most intense in the north–west (Figure 4f) where there was also higher variability in ignition date (Figure S2c). There were pronounced differences in the number of fires and area burned across management units. Whilst the total area burnt and total number of fires are a function of the area of each management unit, management units to the west (SNP, Grumeti, Maswa and Mwiba) had both a greater area burned and number of fires when expressed as a proportion of their land area (Figure S3).

### 3.2 Temporal trends

The number of fires varied between 474 and 1,456 per year, and the area burned varied between 2,819 and 13,017 km^2^ (7.8%–35.9%) per year. The largest 10% of fires accounted for 61.8%–87.2% (median 77.5%) of the area burnt each year. There was a strong positive correlation between the total number of fires and the total area burned each year (*p* < 0.001, *r* ^2^ = 0.82, *df* = 12), no correlation between the median size of the largest 10% of fires and the area burned annually (*p* = 0.63, *r* ^2^ = 0.02, *df* = 12) and no correlation between the median fire size of the largest 10% of fires and the total number of fires annually (*p* = 0.70, *r* ^2^ = 0.01, *df* = 12).

**Figure 5:**
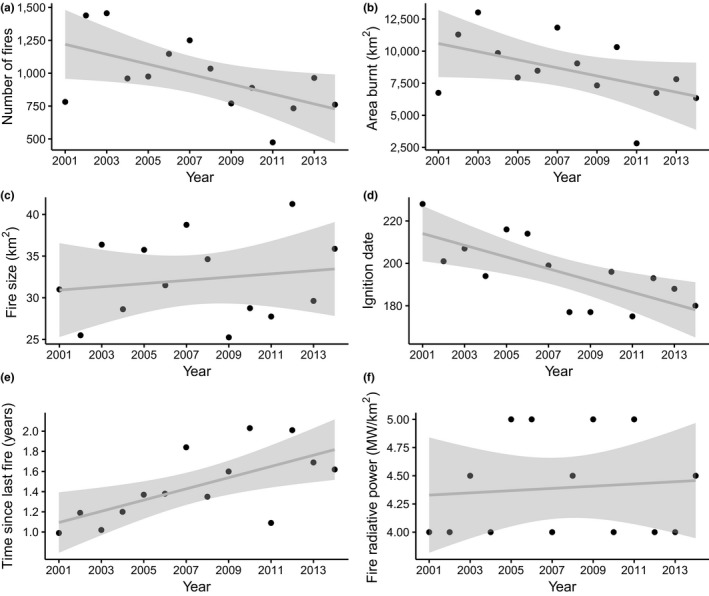
Fitted temporal trends in: (a) total number of fires, (b) total area burnt, (c) median fire size, (d) median ignition date, (e) median time since last fire, (f) median fire radiative power. Linear regression lines are shown in grey and the shaded envelopes represent 95% confidence intervals

There was a 40% decline in the number of fires annually (*p* = 0.03, *r* ^2^ = 0.33, *df* = 12) (Figure 5a) between 2001 and 2014, with a 39% decrease in the area burnt annually (*p* = 0.07, *r* ^2^ = 0.25, *df* = 12) (Figure 5b). Median fire size of the largest 10% of fires (*p* = 0.58, *r* ^2^ = 0.03, *df* = 12) (Figure 5c) and radiative power (*p* = 0.97, *r* ^2^ = 0.0001, *df* = 12) (Figure 5f) did not change over this period, but fires burned earlier in the year (*p* = 0.004, *r* ^2^ = 0.5, *df* = 12) (Figure 5d). The increase in time since last fire (*p* = 0.009, *r* ^2^ = 0.44, *df* = 12) (Figure 5e) is likely an artefact of our methods, as maximum time since last fire increases with study duration.

**Figure 6:**
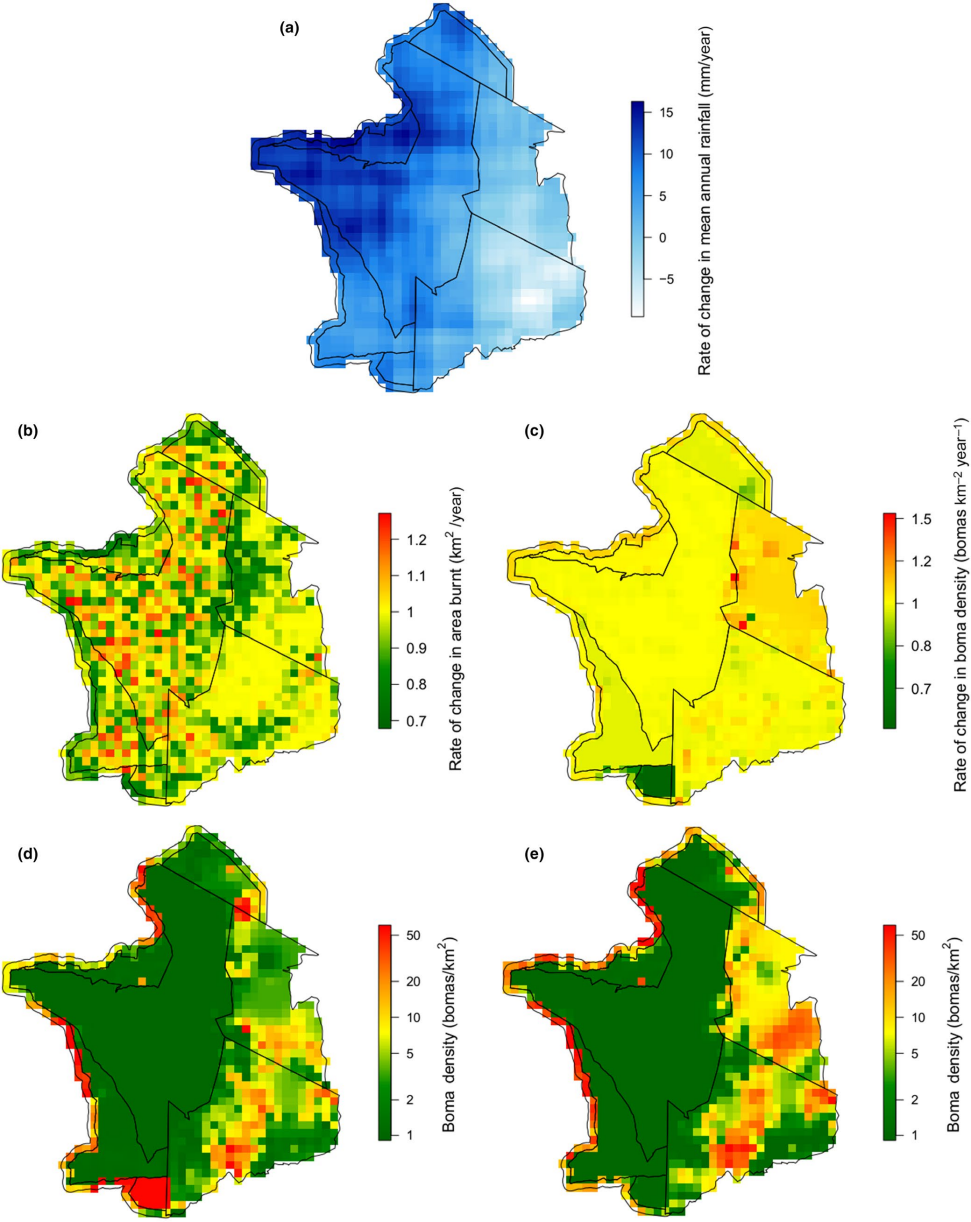
(a) Rate of change in mean annual rainfall (mm/year) from 2001 to 2014, (b) rate of change in area burnt (km^2^/year), (c) rate of change in *boma* density (*bomas *km^−2^ year^−1^), (d) predicted *boma* density in 2001 and (e) predicted *boma* density in 2014. Note active removal of *bomas* from Mwiba (south‐eastern portion of the ecosystem) occurred from 2006

These observed temporal trends differed among management units. The overall decrease in the total area burned was driven by significant reductions in Loliondo, Maasai Mara and the buffer zone (Figure 6b), while the decline in number of fires occurred more widely with only SNP and Maswa showing no significant change. Whilst fire size did not decline across the ecosystem as a whole, there were significant declines in fire size in Ngorongoro and in the buffer zone. Time since last fire increased significantly in all management units except Maswa, and fire radiative power increased significantly in Mwiba (Figure S4).

### 3.3 Spatial drivers of fire

**Figure 7:**
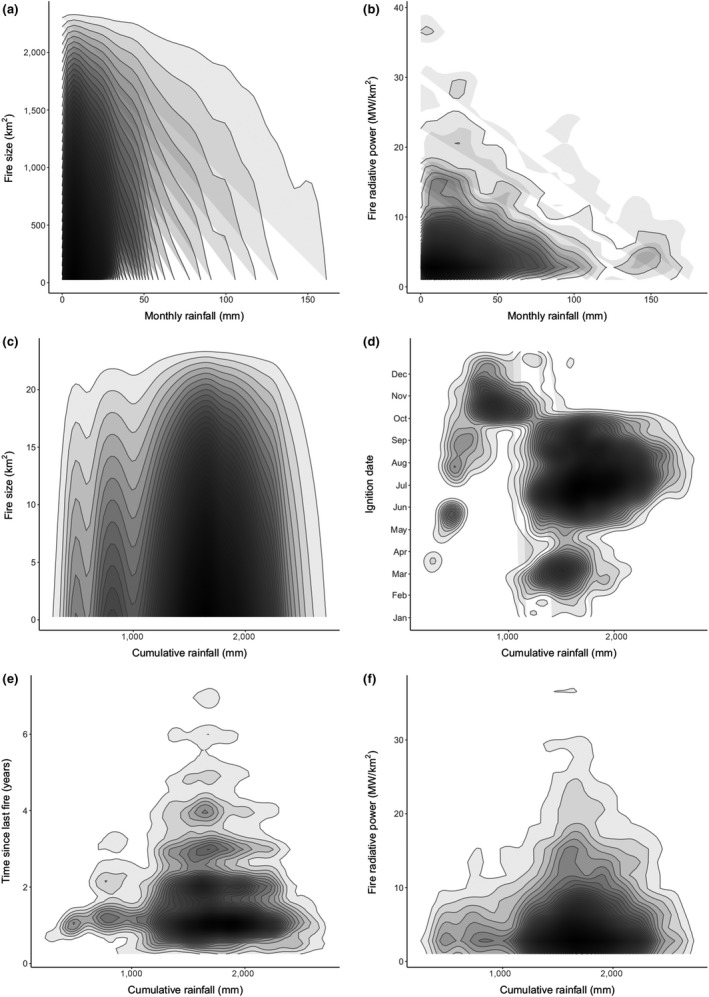
Relationships between monthly rainfall and (a) fire size and (b) fire radiative power, and between cumulative rainfall and (c) fire size, (d) ignition date, (e) time since last fire and (f) fire radiative power. Darker areas indicate the space where the highest density of fires occur

**Figure 8:**
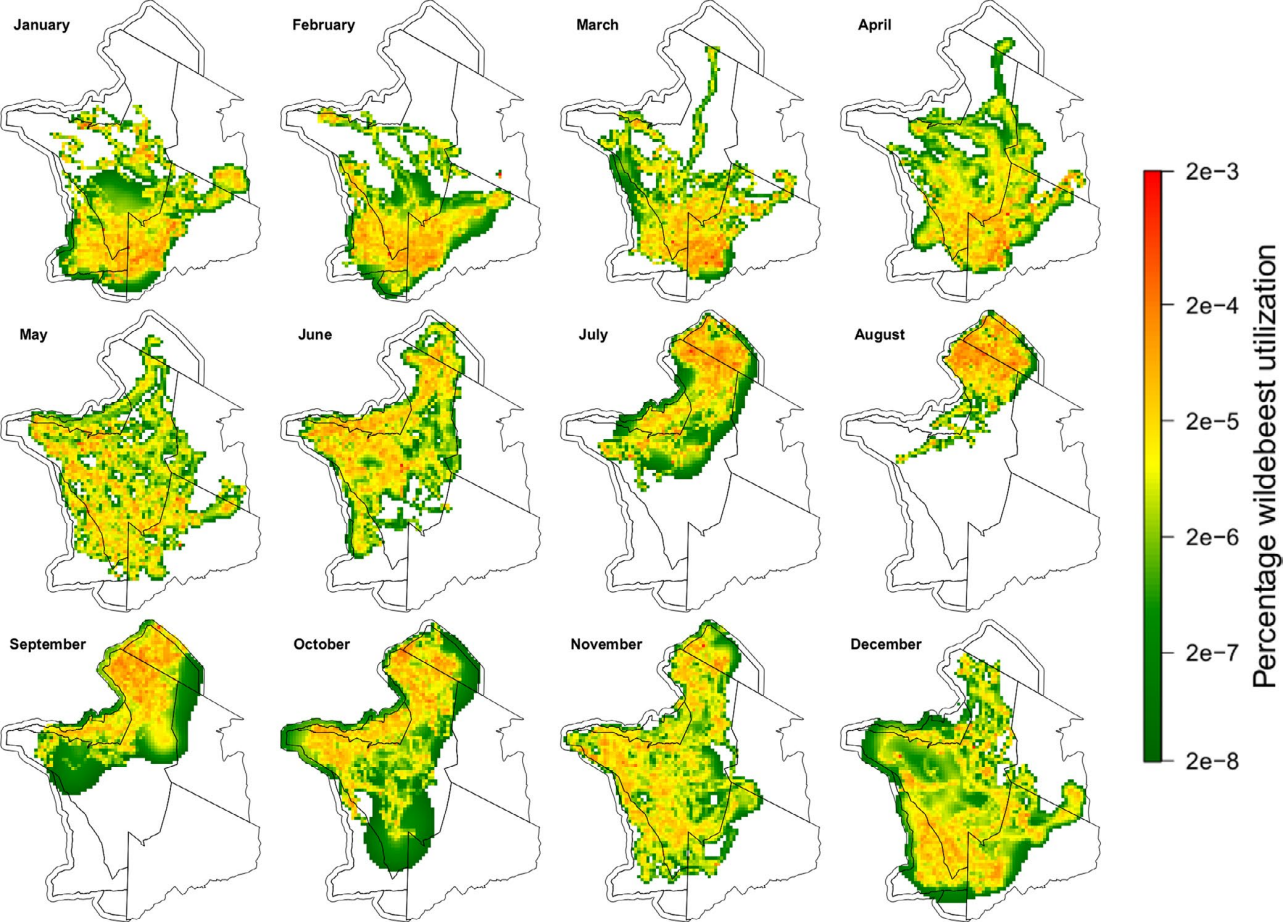
Mean monthly percentage of wildebeest utilization per 500 m^2^

Our models suggest that both monthly and cumulative rainfall drive fire in the Serengeti‐Mara. The results support a link between higher monthly rainfall and smaller fires, monthly NPP (which is linked to monthly rainfall and fuel moisture) with smaller and cooler fires and also suggested higher monthly rainfall shifted fires later into the year (Figure 7; Figure S5). The effect of cumulative rainfall was non‐linear: both high and low cumulative rainfall result in fires occurring later in the year, but also in shorter times since last fire. Our models also suggested that fires were smaller in areas with high *boma* density (Figure 6d,e) and high wildebeest utilization (Figure 8), and detected known methodological issues with MODIS data, such as suggesting that larger fires had higher fire radiative power, and that time since last fire increased with year (Figure S5).

### 3.4 Temporal drivers of fire

We identified 27,145 km^2^ (74.7% of our study area) where two or more satellite images from different years overlapped. Within these areas we recorded 55,940 *bomas* in satellite images dating from 2001 to 2017. The highest *boma* density for an area of overlap in a single year was 56.4 *bomas*/km^2^, while large areas contained no *bomas* throughout the study period. The trends in *boma* density over time differed widely, with some pixels increasing at a rate of 1.53 *bomas* km^−2^ year^−1^ and others decreasing at a rate of 0.55 *bomas* km^−2^ year^−1^ (Figure 6c–e). Verification of the results of our model of *boma* density found a significant positive correlation between predicted and actual *boma* density (*p* = 0.00258, *r* ^2^ = 0.22, *df* = 37), but the ratio of predicted to actual *boma* density was 5:1. A single outlier, which was split almost exactly equally between Maasai Mara and the buffer zone, was excluded from the analysis. We found a significant negative correlation between *boma* density and grass height (*p* = <0.0001, *r* ^2^ = 0.01, *df* = 12,315) and a significant positive correlation between *boma* density and grass growth rates (*p* = <0.0001, *r* ^2^ = 0.008, *df* = 12,315).

**Figure 9:**
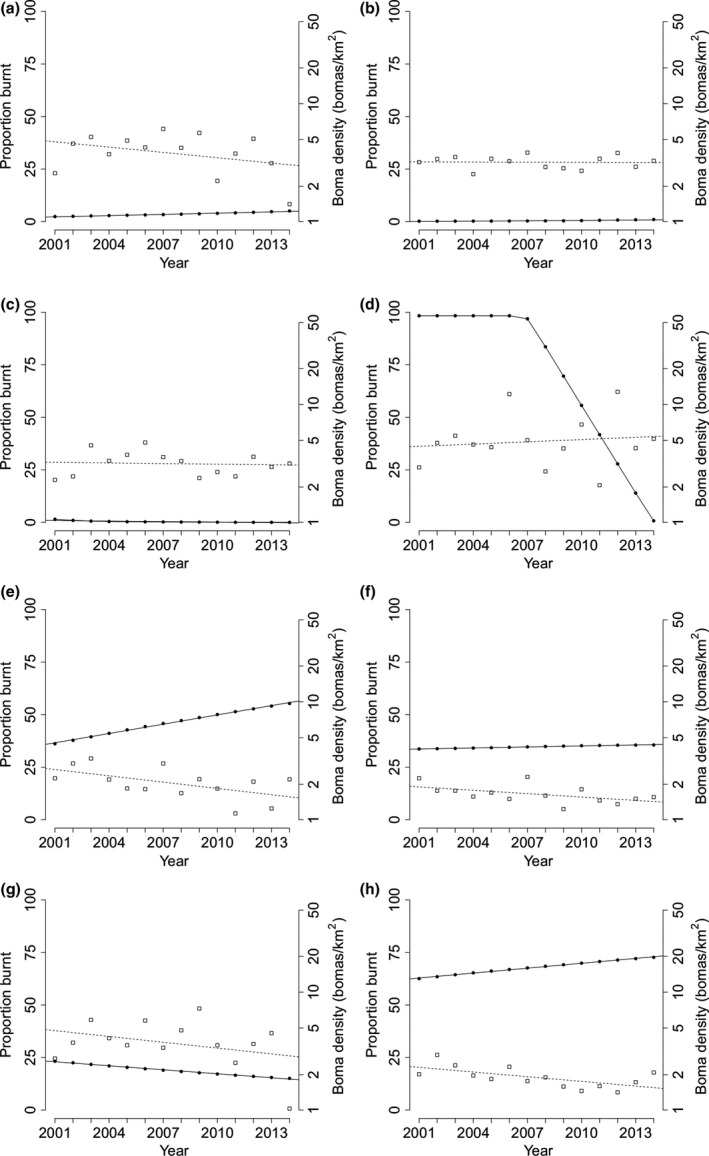
The proportion burnt per year (squares and dotted lines) and the predicted *boma* density per year (circles and solid lines) for: (a) Grumeti, (b) SNP, (c) Maswa, (d) Mwiba, (e) Loliondo, (f) Ngorongoro, (g) Maasai Mara and (h) the buffer zone. Note the contrasting patterns between (d) Mwiba (where *boma* density was decreasing and the area burnt increasing due changes in management), (e) Loliondo (where *boma* density was increasing and the area burnt is decreasing) and (b) SNP (where *boma* density and area burnt were stable)

Our model estimated that an increase of 0.002 *bomas* km^−2^ year^−1^ was associated with a 9.6% decline in the area burned in each cell per year. We found that mean annual rainfall increased in 73% of pixels (Figure 6a) and had a positive effect on the area burnt. A post hoc Tukey's test found no difference in the relationship between area burnt and *boma* density between Grumeti, SNP and Maswa, no difference between NCA, Loliondo and the buffer zone, no difference between Maasai Mara and the buffer zone and no difference between Mwiba and any other management area (Figure 9). The rate of change in area burnt in a pixel was strongly positively correlated with both the rate of change in size (*p* < 0.001, *r* ^2^ = 0.66, *df* = 1,066) and the rate of change in number of fires (*p* < 0.001, *r* ^2^ = 0.42, *df* = 1,066). The rate of change in *boma* density, rate of change in mean annual rainfall, management unit and the baseline area burnt were all significant predictors of the rate of change in the area burnt. There were significant interactions between the rate of change in *boma* density, mean annual rainfall and baseline area burnt, and between the rate of change in mean annual rainfall and mean annual rainfall (Table S2).

## 4 DISCUSSION

We provide the first comprehensive spatial and temporal assessment of fire regimes across the wider Serengeti‐Mara ecosystem. Fire size, season, radiative power and frequency varied across the study area and were strongly linked to differences in rainfall and, to a lesser extent, grazing. Spatial patterns in fire characteristics were highly variable across the Serengeti‐Mara, reflecting the system's strong environmental gradients (Dempewolf et al., 2007). The principal effect of the Serengeti‐Mara's bimodal rainfall pattern generates bimodal fire seasonality with fewer fires in wetter months. Higher monthly rainfall reduced both the size and radiative power of fires occurring in those months, presumably due to the dampening effect of green vegetation and atmospheric moisture on fire. Fires occurred later in areas of both high and low cumulative rainfall. Whilst this result may initially seem contradictory, we suggest that high rainfall areas remain wetter for longer periods, resulting in fires that occur later into the long dry season, whilst low rainfall areas remain dry for longer periods, resulting in fires occurring for longer at the end of the long dry season. Our findings concur with other African studies (e.g. Sinclair, 1975) that conclude that the influence of cumulative rainfall on grass growth rates is primarily responsible for fire size, intensity and return interval (although our study did not make explicit links between rainfall and primary productivity). In contrast, rainfall immediately preceding the date of a fire increases fuel and atmospheric moisture (through surface evaporation and transpiration), decreasing fire size and intensity, and resulting in fewer fires in wet periods (Govender et al., 2006; van Wilgen et al., 2004).

Strikingly, we found that the total area burnt and number of fires declined over time, with some previously burned areas lacking any fire in recent years. This was principally associated with changes in human activities: an increase in livestock density, and variable management objectives and practices. We observed contrasting spatial patterns in the temporal trends that might have been obscured by a larger scale analysis, emphasising that fire studies need to be spatially explicit at the relevant scale. While the positive response to cumulative rainfall was expected (e.g. Archibald et al., 2009), the effect of fire management found here differs from previous studies (e.g. van Wilgen et al., 2004). This raises concerns over the long‐term functioning of the Serengeti‐Mara as a savannah system (Andela et al., 2017; Sinclair et al., 2007) and potential for savannah management regimes to result in modified ecologies.

We proposed several possible drivers of the decreases in the total area burnt: changes in rainfall, changes in management regimes and increased wildebeest numbers. Rainfall increased across most of the ecosystem during our study period and this increase in rainfall had a significant positive effect on the total area burnt (Van Wilgen et al., 2004). However, despite the positive effect of increased rainfall, overall total area burnt still declined. Managers may indirectly affect the fire regime (e.g. by fragmenting the landscape through roads/fire breaks or modifying herbivore distribution through construction of artificial water points) (Beale et al., 2013). They can also have direct impacts, particularly on fire size and season, but the profusion of sources of ignition limits manager's ability to alter the total area burnt (Alvarado, Silva, & Archibald, 2018; Van Wilgen et al., 2004). Van Wilgen et al. (2004) examined fire management approaches in Kruger National Park over a 45 year period and found that total area burnt was a function of rainfall and largely independent of fire management policy, although season of fire could be influenced. We observed fewer fires in the Maasai Mara, suggesting that the policy of fire suppression there is having an effect. However, we did not observe a temporal change in the number of fires or total area burnt in the Maasai Mara, and management in those areas where a decline was observed was not aiming to suppress fire, so it seems unlikely that direct fire suppression by managers could be responsible for such large decreases in the area burnt.

In the 1960s and 1970s the Serengeti‐Mara's wildebeest increased in abundance roughly sixfold, resulting in a reduction in the area burnt through their consumption of grass biomass (Dublin, 1995). Wildebeest abundance stabilized around the mid 1990s (Holdo et al., 2009; Hopcraft et al., 2015), so it is unlikely that an increase in the wildebeest population accounts for the reduction in the area burnt that we observed. It is possible that localized changes in the distribution of wild grazers mediate the fire regime at finer scales (Kimuyu, Sensenig, Riginos, Veblen, & Young, 2014). The organizations managing Grumeti, Maswa and Mwiba have all changed within our study period, leading to changes in management approaches to burning and the intensity of other interventions. In Grumeti there was a fourfold increase in the biomass of resident wild herbivores between 2003 and 2015, including a tenfold increase in its buffalo (*Syncerus caffer* Sparrman, 1779) population (Goodman & Mbise, 2016). This increase is not possible by reproduction alone and must, therefore, be caused by movement of buffalo into Grumeti from other areas of the Serengeti‐Mara, indicating that dispersal may drive local increases and decreases in wild herbivore abundance. However, in Grumeti there was no significant change in any of the six characteristics of the fire regime we examined, suggesting that either the increase in wild herbivore abundance was having no effect, perhaps because the area's high rainfall offsets any impact, or more likely that the effect was being offset by other management actions, such as the exclusion of cattle encroachment and the manipulation of fire ignitions, size and frequency. Without spatially explicit data on wild herbivore abundance it is impossible to test this quantitatively, but it is clear that local shifts in wild herbivore abundance are occurring and it is likely that these shifts and interactions with other ecosystem drivers will have some influence on fire regimes (Goodman & Mbise, 2016).

*Boma* density, and associated livestock density and grazing, was strongly implicated as the key factor driving the decline in the area burnt over our study period. By consuming grass biomass, livestock reduce the available fuel, limiting the ability of fires to spread to the point where they are not large enough to be detected by MODIS (Archibald et al., 2009; Donaldson et al., 2018), and highlighting the importance of fuel loads over ignitions in savannah systems (Archibald et al., 2013; Archibald, Scholes, et al., 2010; Frost, 1999). Our analysis found the relationship between *boma* density and the area burnt differed depending on whether management units excluded (SNP, Grumeti and Maswa) or permitted livestock (NCA, Loliondo and the buffer zone). Any management activity which alters the fuel load will automatically alter fire, whether this is intended or not (e.g. Smit & Archibald, 2019). Our results suggest that management decisions and actions related to livestock may represent the largest effect land managers can have on fire regimes. Previous studies in the Serengeti‐Mara observed a decline in the area burnt as wild herbivore numbers increased (Dublin, 1995); it is noteworthy that the decline we observed took place within the Serengeti‐Mara's protected areas and was caused by increasing populations of relatively sedentary livestock, rather than seasonal grazing by migratory wild herbivores. Our findings also contrast previous studies which found that land‐cover change was restricted primarily to the Kenyan sector of the Serengeti‐Mara (Homewood et al., 2001).

Overgrazing is politically loaded term frequently cited as a cause of degradation in savannah and grassland ecosystems (Brauch & Spring, 2009; Oldeman, Hakkeling, & Sombroek, 1990), leading to reduced biodiversity (Alkemade, Reid, Berg, Leeuw, & Jeuken, 2013), increased soil erosion (Kosmas et al., 2015), decreased soil carbon storage (Dlamini, Chivenge, & Chaplot, 2016), bush encroachment (Coetzee, Tincani, Wodu, & Mwasi, 2008) and desertification (Homewood & Rodgers, 1987). We can add the modification of fire regimes, and specific characteristics of fire, to this list (Archibald et al., 2012; Hempson et al., 2018). In the buffer zone and parts of Ngorongoro and Loliondo the area burnt has been reduced to virtually zero. This exclusion of fire represents a substantial shift in the dominant driver of spatial heterogeneity in these areas, a shift which is outside the range of variation with which this system evolved (Gillson & Duffin, 2007), and which may surpass critical ecological thresholds (Gillson & Ekblom, 2009) and lead to a change in stable state (Eby, Agrawal, Majumder, Dobson, & Guttal, 2017). Fire plays an important role in governing the structure and function of the Serengeti‐Mara (Anderson et al., 2007; Holdo et al., 2009) and its exclusion could lead to an increase in bush encroachment (O'Connor, Puttick, & Hoffman, 2014) and the displacement of wild herbivores (Madhusudan, 2004). This will simultaneously limit productivity in terms of pastoralism and tourism; the two principal means of income generation in the region. Existing livestock densities in some areas may be too high for savannahs to persist in their current state. There is, therefore, an urgent need to re‐evaluate the condition of and approaches to management for these areas to ensure the success of both conservation objectives and the socio‐economic prosperity of the Serengeti‐Mara's human population.

This is the first study to document the spatial and temporal fire patterns and drivers across the wider Serengeti‐Mara ecosystem. Our findings are consistent with studies reporting a global decline in the area burnt and raising concerns about the impact this decline has on the ecology of these ecosystems (Andela et al., 2017). The suppression of fire is likely to result in changes in the structure, function and biodiversity of some areas of the Serengeti‐Mara which may not be compatible with the objectives of the stakeholders involved in these areas (Trollope, Trollope, & Hartnett, 2002). For instance, a possible consequence of a decline in the area burnt is the increase in understorey tree and bush recruitment recently documented in the Serengeti‐Mara (Holdo, Anderson, & Morrison, 2014; O'Connor et al., 2014). Our results suggest that some areas of the Serengeti‐Mara may already have been substantially modified and the widespread decreasing trend in the number of fires and the area burnt indicates other areas require close monitoring to achieve the desired management outputs. If periodic burning is incorporated into an explicit management plan, then our results suggest that altering the fire‐suppressive effects of intense grazing by resident livestock will result in more fires and a larger total area burnt. Our findings underscore the importance of managers monitoring fire, using the information they gather to inform future management decisions and to develop fire regimes that promote management objectives and contribute to the spatiotemporal resiliency of the savannah ecosystems in the region.

## References

[gcb14711-bib-0001] Alkemade, R. , van den Reid, R. S. , de Berg, M. , Leeuw, J. , & Jeuken, M. (2013). Assessing the impacts of livestock production on biodiversity in rangeland ecosystems. Proceedings of the National Academy of Sciences of the United States of America, 110(52), 20900–20905. 10.1073/pnas.1011013108 22308313 PMC3876242

[gcb14711-bib-0002] Alvarado, S. T. , Silva, T. S. F. , & Archibald, S. (2018). Management impacts on fire occurrence: A comparison of fire regimes of African and South American tropical savannas in different protected areas. Journal of Environmental Management, 15(218), 79–87. 10.1016/j.jenvman.2018.04.004 29665489

[gcb14711-bib-0003] Andela, N. , Morton, D. C. , Giglio, L. , Chen, Y. , van der Werf, G. R. , Kasibhatla, P. S. , … Randerson, J. T. (2017). A human‐driven decline in global burned area. Science, 356(6345), 1356–1362. 28663495 10.1126/science.aal4108 PMC6047075

[gcb14711-bib-0004] Anderson, T. M. , Ritchie, M. E. , Mayemba, E. , Eby, S. L. , Grace, J. B. , & McNaughton, S. J. (2007). Forage nutritive quality in the Serengeti ecosystem: The roles of fire and herbivory. The American Naturalist, 170(3), 343–357. 10.1086/520120 17879186

[gcb14711-bib-0005] Anderson, G. D. , & Talbot, L. M. (1965). Soil factors affecting the distribution of the grassland types and their utilization by wild animals on the Serengeti plains, Tanganyika. Journal of Ecology, 53(1), 33–56. 10.2307/2257564

[gcb14711-bib-0006] Archibald, S. , Lehmann, C. E. R. , Gomez-Dans, J. L. , & Bradstock, R. A. (2013). Defining pyromes and global syndromes of fire regimes. Proceedings of the National Academy of Sciences of the United States of America, 110(16), 6442–6447. 10.1073/pnas.1211466110 23559374 PMC3631631

[gcb14711-bib-0007] Archibald, S. , Nickless, A. , Govender, N. , Scholes, R. J. , & Lehsten, V. (2010). Climate and the inter‐annual variability of fire in southern Africa: A meta‐analysis using long‐term field data and satellite‐derived burnt area data. Global Ecology and Biogeography, 19(6), 794–809. 10.1111/j.1466-8238.2010.00568.x

[gcb14711-bib-0008] Archibald, S. , Scholes, R. J. , Roy, D. P. , Roberts, G. , & Boschetti, L. (2010). Southern African fire regimes as revealed by remote sensing. International Journal of Wildland Fire, 19(7), 861–878. 10.1071/WF10008

[gcb14711-bib-0009] Archibald, S. , Staver, A. C. , & Levin, S. A. (2012). Evolution of human‐driven fire regimes in Africa. Proceedings of the National Academy of Sciences of the United States of America, 109(3), 847–852. 10.1073/pnas.1118648109 22184249 PMC3271907

[gcb14711-bib-0010] Archibald, S. , van Roy, D. P. , Wilgen, B. W. , & Scholes, R. J. (2009). What limits fire? An examination of drivers of burnt area in Southern Africa. Global Change Biology, 15(3), 613–630. 10.1111/j.1365-2486.2008.01754.x

[gcb14711-bib-0011] Balfour, D. , & Howison, O. (2002). Spatial and temporal variation in a mesic savanna fire regime: Responses to variation in annual rainfall. African Journal of Range & Forage Science, 19(1), 45–53. 10.2989/10220110209485773

[gcb14711-bib-0012] Beale, C. M. , Courtney Mustaphi, C. J. , Morrison, T. A. , Archibald, S. , Anderson, T. M. , Dobson, A. P. , … Parr, C. L. (2018). Pyrodiversity interacts with rainfall to increase bird and mammal richness in African savannas. Ecology Letters, 21, 557–567. 10.1111/ele.12921 29441661 PMC5888149

[gcb14711-bib-0013] Beale, C. M. , Lennon, J. J. , Yearsley, J. M. , Brewer, M. J. , & Elston, D. A. (2010). Regression analysis of spatial data. Ecology Letters, 13(2), 246–264. 10.1111/j.1461-0248.2009.01422.x 20102373

[gcb14711-bib-0014] Beale, C. M. , Rensberg, S. V. , Bond, W. J. , Coughenour, M. , Fynn, R. , Gaylard, A. , … Sinclair, A. R. E. (2013). Ten lessons for the conservation of African savannah ecosystems. Biological Conservation, 167, 224–232. 10.1016/j.biocon.2013.08.025

[gcb14711-bib-0015] Bond, W. J. (2005). Large parts of the world are brown or black: A different view on the “Green World” hypothesis. Journal of Vegetation Science, 16(3), 261–266. 10.1658/1100-9233(2005)016[0261:LPOTWA]2.0.CO;2

[gcb14711-bib-0016] Bond, W. J. , & Keeley, J. E. (2005). Fire as a global ‘herbivore’: The ecology and evolution of flammable ecosystems. Trends in Ecology & Evolution, 20(7), 387–394. 10.1016/j.tree.2005.04.025 16701401

[gcb14711-bib-0017] Bond, W. J. , Woodward, F. I. , & Midgley, G. F. (2005). The Global Distribution of Ecosystems in a world without Fire. New Phytologist, 165(2), 525–538. 10.1111/j.1469-8137.2004.01252.x 15720663

[gcb14711-bib-0018] Bowman, D. M. J. S. , Balch, J. K. , Artaxo, P. , Bond, W. J. , Carlson, J. M. , Cochrane, M. A. , & Pyne, S. J. (2009). Fire in the earth system. Science, 324(5926), 481–484. 19390038 10.1126/science.1163886

[gcb14711-bib-0019] Bowman, D. M. J. S. , Balch, J. , Artaxo, P. , Bond, W. J. , Cochrane, M. A. , D'Antonio, C. M. , … Swetnam, T. W. (2011). The human dimension of fire regimes on Earth. Journal of Biogeography, 38(12), 2223–2236. 10.1111/j.1365-2699.2011.02595.x 22279247 PMC3263421

[gcb14711-bib-0020] Brauch, H. G. , & Spring, U. O. (2009). Securitizing the ground grounding security UNCCD issue paper No. 2, Secretariat of the United Nations Convention to Combat Desertification, Bonn.

[gcb14711-bib-0021] Buthelezi, N. L. S. , Mutanga, O. , Rouget, M. , & Sibanda, M. (2016). A spatial and temporal assessment of fire regimes on different vegetation types using MODIS burnt area products. Bothalia, 46(2), 1–9. 10.4102/abc.v46i2.2148

[gcb14711-bib-0022] Coetzee, B. W. , Tincani, L. , Wodu, Z. , & Mwasi, S. M. (2008). Overgrazing and bush encroachment by *Tarchonanthus camphoratus* in a semi‐arid savanna. African Journal of Ecology, 46, 449–451. 10.1111/j.1365-2028.2007.00842.x

[gcb14711-bib-0023] Copernicus . (2018). Copernicus Sentinel data 2016 & 2017. Retrieved from GEE 10th December 2018, processed by ESA.

[gcb14711-bib-0024] Cressie, N. (1993). Statistics for spatial data, revised edition. New York, NY: John Wiley and Sons, Inc.

[gcb14711-bib-0025] Dempewolf, J. , Trigg, S. , DeFries, R. S. , & Eby, S. (2007). Burned-area mapping of the Serengeti–Mara region using MODIS reflectance data. IEEE Geoscience and Remote Sensing Letters, 4(2), 312–316. 10.1109/LGRS.2007.894140

[gcb14711-bib-0026] Dlamini, P. , Chivenge, P. , & Chaplot, V. (2016). Overgrazing decreases soil organic carbon stocks the most under dry climates and low soil pH: A meta‐analysis shows. Agriculture, Ecosystems and Environment, 221, 258–269. 10.1016/j.agee.2016.01.026

[gcb14711-bib-0027] Donaldson, J. E. , Archibald, S. , Govender, N. , Pollard, D. , Luhdo, Z. , & Parr, C. L. (2018). Ecological engineering through fire‐herbivory feedbacks drives the formation of savanna grazing lawns. Journal of Applied Ecology, 55, 225–235. 10.1111/1365-2664.12956

[gcb14711-bib-0028] Dublin, H. T. (1995). Vegetation dynamics in the Serengeti–Mara Ecosystem: The role of elephants, fire and other factors In Sinclair A. R. E. & Arcese P. (Eds.), Serengeti II – Dynamics, management and conservation of an ecosystem (pp. 71–90). London: University of Chicago Press.

[gcb14711-bib-0029] Eby, S. , Agrawal, A. , Majumder, S. , Dobson, A. P. , & Guttal, V. (2017). Alternative stable states and spatial indicators of critical slowing down along a spatial gradient in a savanna ecosystem. Global Ecology and Biogeography, 2017(26), 638–649. 10.1111/geb.12570

[gcb14711-bib-0030] Estes, A. B. , Kuemmerle, T. , Kushnir, H. , Radeloff, V. C. , & Shugart, H. H. (2012). Land‐cover change and human population trends in the greater Serengeti ecosystem from 1984–2003. Biological Conservation, 147(1), 255–263. 10.1016/j.biocon.2012.01.010

[gcb14711-bib-0031] Fischer, G. F. , Nachtergaele, S. , van Prieler, H. T. , Velthuizen, L. , & Verelst, D. W. (2008). Global agro‐ecological zones assessment for agriculture. Laxenburg, Austria/Rome, Italy: IIASA/FAO.

[gcb14711-bib-0032] Frost, P. G. H. (1999). Fire in southern African woodlands: Origins, impacts, effects, and control. Proceedings of an FAO Meeting on Public Policies Affecting Forest Fires, 138(January), 181–205.

[gcb14711-bib-0033] Frost, P. G. H. , & Robertson, F. (1987). The ecological effects of fire in savannas In Walker B. H. (Ed.), Determinants of tropical savannas (pp. 93–140). Miami, FL: ICSU Press.

[gcb14711-bib-0034] Funk, C. , Peterson, P. , Landsfeld, M. , Pedreros, D. , Verdin, D. , Shukla, S. , … Michaelsen, J. (2015). The climate hazards infrared precipitation with stations—A new environmental record for monitoring extremes. Scientific Data, 2, 150066 10.1038/sdata.2015.66 26646728 PMC4672685

[gcb14711-bib-0035] Gill, A. M. (1975). Fire and the Australian flora: A review. Australian Forestry, 38, 4–25. 10.1080/00049158.1975.10675618

[gcb14711-bib-0036] Gillson, L. , & Duffin, K. (2007). Thresholds of potential concern as benchmarks in the management of African savannahs. Philosophical Transactions of the Royal Society B: Biological Sciences, 362(1478), 309–319. 10.1098/rstb.2006.1988 PMC2311432 17255038

[gcb14711-bib-0037] Gillson, L. , & Ekblom, A. (2009). Resilience and thresholds in savannas: Nitrogen and fire as drivers and responders of vegetation transition. Ecosystems, 12, 1189 10.1007/s10021-009-9284-y

[gcb14711-bib-0038] Goodman, P. S. , & Mbise, N. L. (2016). Large herbivore population estimates for the Grumeti Reserves – August 2014.

[gcb14711-bib-0039] Google Earth . (2017). Serengeti, Tanzania. DigitalGlobe.

[gcb14711-bib-0040] Gorelick, N. , Hancher, M. , Dixon, M. , Ilyushchenko, S. , Thau, D. , & Moore, R. (2017). Google Earth Engine: Planetary‐scale geospatial analysis for everyone. Remote Sensing of Environment, 202, 18–27. 10.1016/j.rse.2017.06.031

[gcb14711-bib-0041] Govender, N. , Trollope, W. S. W. , & Van Wilgen, B. W. (2006). The effect of fire season, fire frequency, rainfall and management on fire intensity in savanna vegetation in South Africa. Journal of Applied Ecology, 43(4), 748–758. 10.1111/j.1365-2664.2006.01184.x

[gcb14711-bib-0042] Guyette, R. P. , Muzika, R. M. , & Dey, D. C. (2002). Dynamics of an anthropogenic fire regime. Ecosystems, 5(5), 472–486.

[gcb14711-bib-0043] Hempson, G. P. , Parr, C. L. , Archibald, S. , Anderson, T. M. , Mustaphi, C. J. C. , Dobson, A. P. , … Beale, C. M. (2018). Continent‐level drivers of African pyrodiversity. Ecography, 41, 889–899.

[gcb14711-bib-0044] Holdo, R. M. , Anderson, T. M. , & Morrison, T. A. (2014). Precipitation, fire and demographic bottleneck dynamics in Serengeti tree populations. Landscape Ecology, 29, 1613–1623. 10.1007/s10980-014-0087-y

[gcb14711-bib-0045] Holdo, R. M. , Holt, R. D. , & Fryxell, J. M. (2009). Grazers, browsers, and fire influence the extent and spatial pattern of tree cover in the Serengeti. Ecological Applications, 19(1), 95–109. 10.1890/07-1954.1 19323175

[gcb14711-bib-0046] Homewood, K. , Lambin, E. F. , Coast, E. , Kariuki, A. , Kikula, I. , Kivelia, J. , … Thompson, M. (2001). Long‐term changes in Serengeti‐Mara wildebeest and land cover: Pastoralism, population, or policies? Proceedings of the National Academy of Sciences of the United States of America, 98(22), 12544–12549. 10.1073/pnas.221053998 11675492 PMC60090

[gcb14711-bib-0047] Homewood, K. , & Rodgers, W. A. (1987). Pastoralism, conservation and the overgrazing controversy In Anderson D. & Grove R. (Eds.), Conservation in Africa: People, policies, and practice (pp. 111‐128). Cambridge: Cambridge University Press.

[gcb14711-bib-0048] Hopcraft, G. , Sinclair, A. R. E. , Holdo, M. , Mwangomo, E. , Mduma, S. , Thirgood, S. , & Olff, H. (2015). Why are wildebeest the most abundant herbivore in the Serengeti ecosystem? In Sinclair A. R. E., Metzger K. L., Mduma S. A. R., & Fryxell J. M. (Eds.) Serengeti IV: Sustaining biodiversity in a coupled human‐natural system (pp. 35–71). Chicago, IL: University of Chicago Press.

[gcb14711-bib-0049] Hopcraft, J. G. C. , Morales, J. M. , Beyer, D. E. , Borner, M. , Mwangomo, E. , Sinclair, A. R. E. , … Haydon, D. T. (2014). Competition, predation, and migration: Individual choice patterns of Serengeti migrants captured by hierarchical models. Ecological Monographs, 84, 355–372. 10.1890/13-1446.1

[gcb14711-bib-0050] Horne, J. S. , Garton, E. O. , Krone, S. M. , & Lewis, J. S. (2007). Analyzing animal movements using Brownian Bridges. Ecology, 88, 2354–2363. 10.1890/06-0957.1 17918412

[gcb14711-bib-0051] Kimuyu, D. M. , Sensenig, R. L. , Riginos, C. , Veblen, K. E. , & Young, T. P. (2014). Native and domestic browsers and grazers reduce fuels, fire temperatures, and acacia ant mortality in an African savanna. Ecological Applications, 24(4), 741–749. 10.1890/13-1135.1 24988772

[gcb14711-bib-0052] Kosmas, C. , Detsis, V. , Karamesouti, M. , Kounalaki, K. , Vassiliou, P. , & Salvati, L. (2015). Exploring long‐term impact of grazing management on land degradation in the socio‐ecological system of Asteroussia Mountains, Greece. Land, 4(3), 541–559. 10.3390/land4030541

[gcb14711-bib-0053] Krawchuk, M. A. , & Moritz, M. A. (2014). Burning issues: Statistical analyses of global fire data to inform assessments of environmental change. Environmetrics, 25(6), 472–481.

[gcb14711-bib-0054] Laris, P. (2002). Burning the seasonal mosaic: Preventative burning strategies in the wooded savanna of southern Mali. Human Ecology, 30(2), 155–186.

[gcb14711-bib-0055] Le Page, Y. , Oom, D. , Silva, J. M. N. , Jönsson, P. , & Pereira, J. M. C. (2010). Seasonality of vegetation fires as modified by human action: Observing the deviation from eco‐climatic fire regimes. Global Ecology and Biogeography, 19(4), 575–588. 10.1111/j.1466-8238.2010.00525.x

[gcb14711-bib-0056] Lindgren, F. , Rue, H. , & Lindström, J. (2011). An explicit link between Gaussian fields and Gaussian Markov random fields: The stochastic partial differential equation approach. Journal of the Royal Statistical Society, Series B, 73, 423–498. 10.1111/j.1467-9868.2011.00777.x

[gcb14711-bib-0057] Madhusudan, M. D. (2004). Recovery of wild large herbivores following livestock. Journal of Applied Ecology, 41, 858–869.

[gcb14711-bib-0058] Martins, T. G. , Simpson, D. , Lindgren, F. , & Rue, H. (2013). Bayesian computing with INLA: New features. Computational Statistics and Data Analysis, 67(2009), 68–83.

[gcb14711-bib-0059] Meyer, D. (2011). ASTER global digital elevation model version 2 – Summary of validation results. NASA Land Processes Distributed Active Archive Center and the Joint Japan‐US ASTER Science Team.

[gcb14711-bib-0060] O'Connor, T. G. , Puttick, J. R. , & Hoffman, M. T. (2014). Bush encroachment in southern Africa: Changes and causes. African Journal of Range & Forage Science, 31(2), 67–88. 10.2989/10220119.2014.939996

[gcb14711-bib-0061] Ogutu, J. O. , Bhola, N. , Piepho, H. P. , & Reid, R. (2006). Efficiency of strip‐ and line‐transect surveys of African savanna mammals. Journal of Zoology, 269(2), 149–160. 10.1111/j.1469-7998.2006.00055.x

[gcb14711-bib-0062] Oldeman, L. R. , Hakkeling, R. T. A. , & Sombroek, W. (1990). World map of the status of human‐induced soil degradation: An explanatory note. Working Paper 90/07. Wageningen: Global Assessment of Soil Degradation (GLASOD), ISRIC.

[gcb14711-bib-0063] Parr, C. L. , & Brockett, B. H. (1999). Patch‐mosaic burning: A new paradigm for savanna fire management in protected areas? Koedoe, 42(2), 117–130. 10.4102/koedoe.v42i2.237

[gcb14711-bib-0064] Parr, C. L. , Lehmann, C. E. R. , Bond, W. J. , Hoffmann, W. A. , & Andersen, A. N. (2014). Tropical grassy biomes: Misunderstood, neglected, and under threat. Trends in Ecology & Evolution, 29(4), 205–213. 10.1016/j.tree.2014.02.004 24629721

[gcb14711-bib-0065] Parr, C. L. , Robertson, H. G. , Biggs, H. C. , & Chown, S. L. (2004). Response of African savanna ants to long‐term fire regimes. Journal of Applied Ecology, 41(1), 630–642. 10.1111/j.0021-8901.2004.00920.x

[gcb14711-bib-0066] Pausas, J. G. , & Bradstock, R. A. (2007). Fire persistence traits of plants along a productivity and disturbance gradient in mediterranean shrublands of south‐east Australia. Global Ecology and Biogeography, 16(3), 330–340. 10.1111/j.1466-8238.2006.00283.x

[gcb14711-bib-0067] Pyne, S. J. , Andrews, P. L. , & Laven, R. D. (1996). Introduction to wildland fire (2nd ed.). New York, NY: Wiley.

[gcb14711-bib-0068] R Core Team . (2012). R: A language and environment for statistical computing. Vienna, Austria: R Foundation for Statistical Computing.

[gcb14711-bib-0069] Rue, H. , Martino, S. , & Chopin, N. (2009). Approximate Bayesian inference for latent Gaussian models by using integrated nested Laplace approximations. Journal of the Royal Statistical Society. Series B: Statistical Methodology, 71(2), 319–392. 10.1111/j.1467-9868.2008.00700.x

[gcb14711-bib-0070] Running, S. W. , Nemani, R. R. , Heinsch, F. A. , Zhao, M. , Reeves, M. , & Hashimoto, H. (2004). A continuous satellite‐derived measure of global terrestrial primary production. BioScience, 54(6), 547 10.1641/0006-3568(2004)054[0547:ACSMOG]2.0.CO;2

[gcb14711-bib-0071] Running, S. W. , & Zhao, M. (2015). Daily GPP and annual NPP (MOD17A2/A3) products NASA earth observing system MODIS land algorithm – User's guide V3, 28.

[gcb14711-bib-0072] Sawyer, H. , Kauffman, M. J. , Nielson, R. M. , & Horne, J. S. (2009). Identifying and prioritizing ungulate migration routes for landscape‐level conservation. Ecological Applications, 19, 2016–2025. 10.1890/08-2034.1 20014575

[gcb14711-bib-0073] Sinclair, A. R. E. (1975). The resource limitation of trophic levels in tropical grassland ecosystems. Journal of Animal Ecology, 44(2), 497–520. 10.2307/3608

[gcb14711-bib-0074] Sinclair, A. R. E. , Mduma, S. A. R. , Hopcraft, J. G. C. , Fryxell, J. M. , Hilborn, R. , & Thirgood, S. (2007). Long‐term ecosystem dynamics in the Serengeti: Lessons for conservation. Conservation Biology, 21(3), 580–590. 10.1111/j.1523-1739.2007.00699.x 17531037

[gcb14711-bib-0075] Smit, I. P. J. , & Archibald, S. (2019). Herbivore culling influences spatio‐temporal patterns of fire in a semiarid savanna. Journal of Applied Ecology, 56, 711–721. 10.1111/1365-2664.13312

[gcb14711-bib-0076] Smit, I. P. J. , Smit, C. F. , Govender, N. , van der Linde, M. , & MacFadyen, S. (2013). Rainfall, geology and landscape position generate large‐scale spatiotemporal fire pattern heterogeneity in an African savanna. Ecography, 36, 447–459. 10.1111/j.1600-0587.2012.07555.x

[gcb14711-bib-0077] Tarimo, B. , Dick, Ø. B. , Gobakken, T. , & Totland, Ø. (2015). Spatial distribution of temporal dynamics in anthropogenic fires in miombo savanna woodlands of Tanzania. Carbon Balance and Management, 10(1), 18 10.1186/s13021-015-0029-2 26246851 PMC4518077

[gcb14711-bib-0078] Trollope, W. S. W. , Trollope, L. A. , & Hartnett, D. C. (2002). Fire behaviour a key factor in the fire ecology of African grasslands and savannas In Forest fire research & wildland fire safety (pp. 1–15). Amsterdam, the Netherlands: IOS Press.

[gcb14711-bib-0079] Van wilgen, B. W. W. , Govender, N. , Biggs, H. C. C. , Ntsala, D. , & Funda, X. N. N. (2004). Response of savanna fire regimes to changing fire‐management policies in a large African National Park. Conservation Biology, 18(6), 1533–1540. 10.1111/j.1523-1739.2004.00362.x

[gcb14711-bib-0080] Veblen, K. E. (2013). Impacts of traditional livestock corrals on woody plant communities in an East African savanna. Rangeland Journal, 35(3), 349–353. 10.1071/RJ13001

[gcb14711-bib-0081] Williams, R. J. L. B. , Hutley, G. D. , Cook, J. , Russell‐Smith, A. E. , & Chen, X. (2004). Assessing the carbon sequestration potential of mesic savannas in the Northern Territory, Australia: Approaches, uncertainties and potential impacts of fire. Functional Plant Biology, 31(5), 415–422. 10.1071/FP03215 32688913

[gcb14711-bib-0082] Wood, S. W. , Murphy, B. P. , & Bowman, D. M. J. S. (2011). Firescape ecology: How topography determines the contrasting distribution of fire and rain forest in the south‐west of the Tasmanian Wilderness World Heritage Area. Journal of Biogeography, 38(9), 1807–1820. 10.1111/j.1365-2699.2011.02524.x

