# Supplementary figures and images for: Anthropogenic modifications to fire regimes in the wider Serengeti‐Mara ecosystem
Source: Glob Chang Biol. 2019 Jul 8;25(10):3406–23. doi: 10.1111/gcb.14711 (PMC6852266; doi:10.1111/gcb.14711)

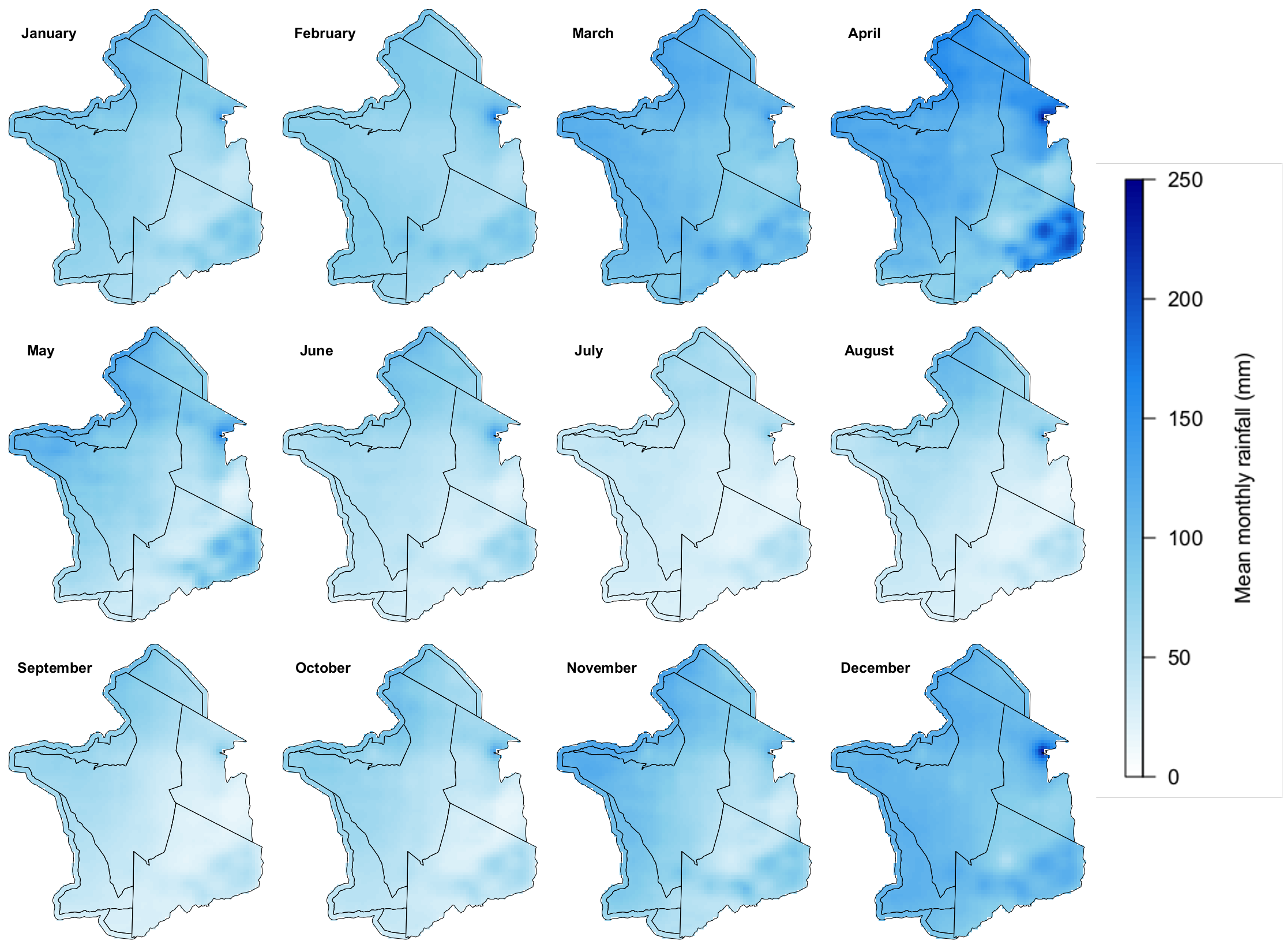

Supplement: Supplementary file 1 [file GCB-25-3406-s001.tiff]

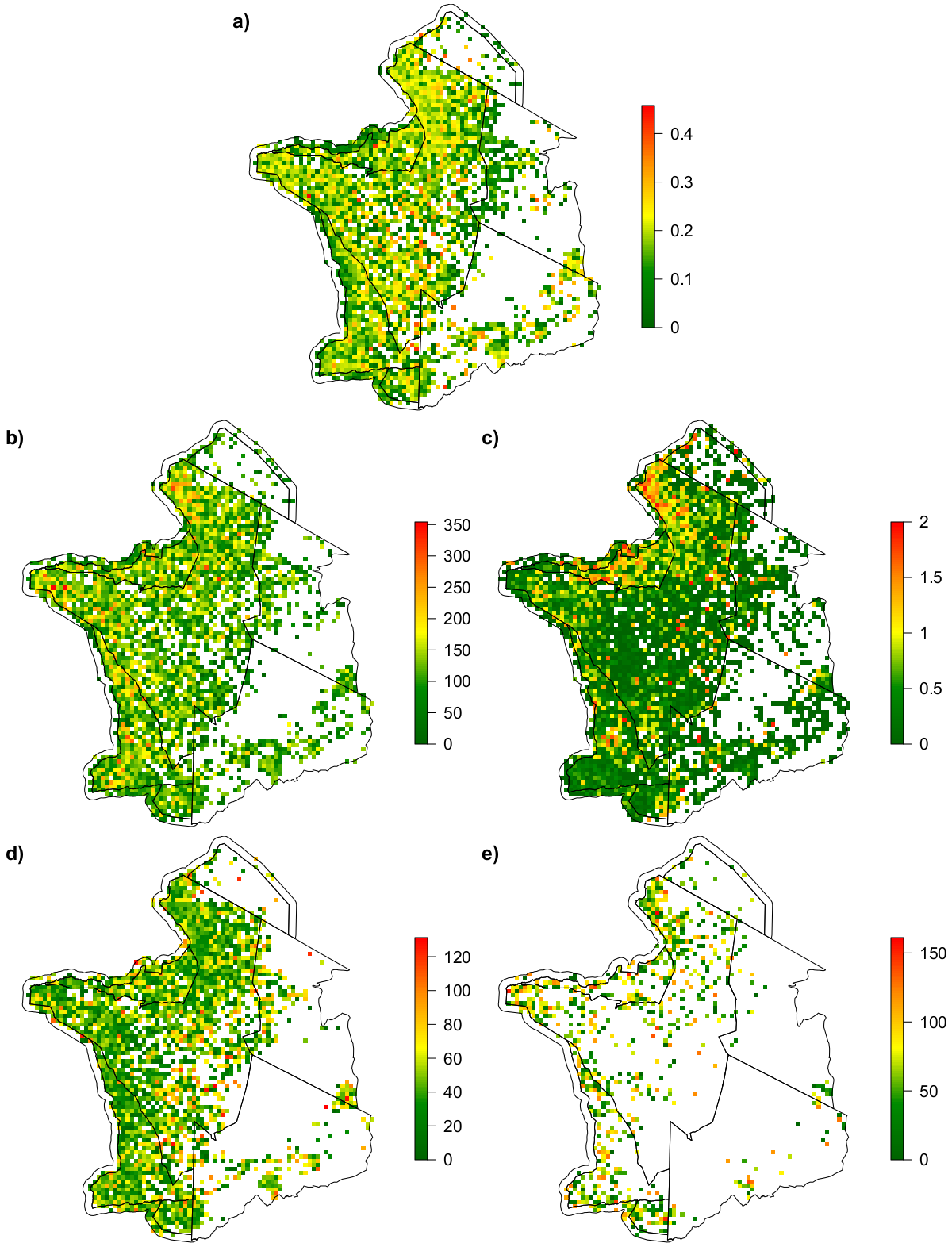

Supplement: Supplementary file 2 [file GCB-25-3406-s002.TIFF]

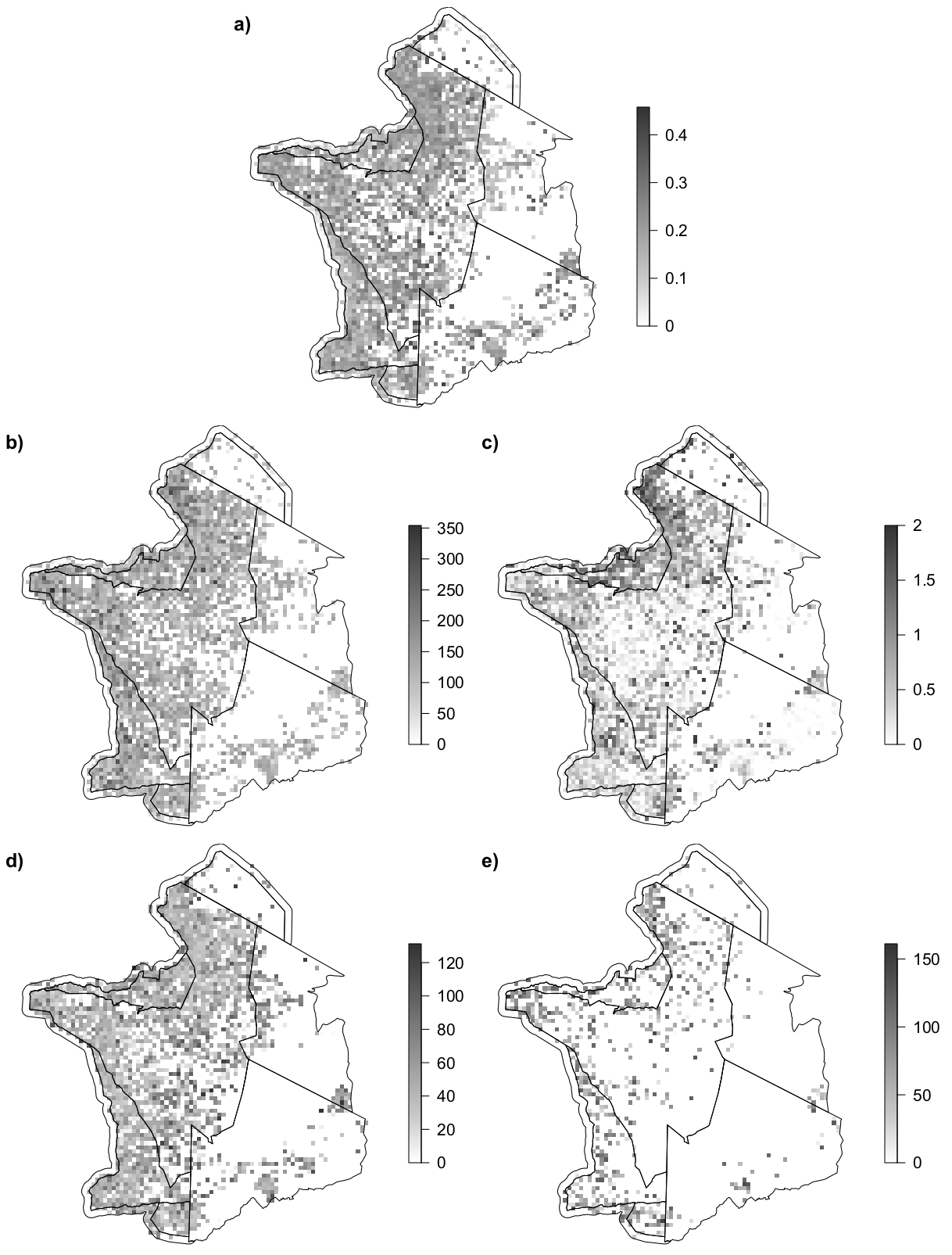

Supplement: Supplementary file 3 [file GCB-25-3406-s003.TIFF]

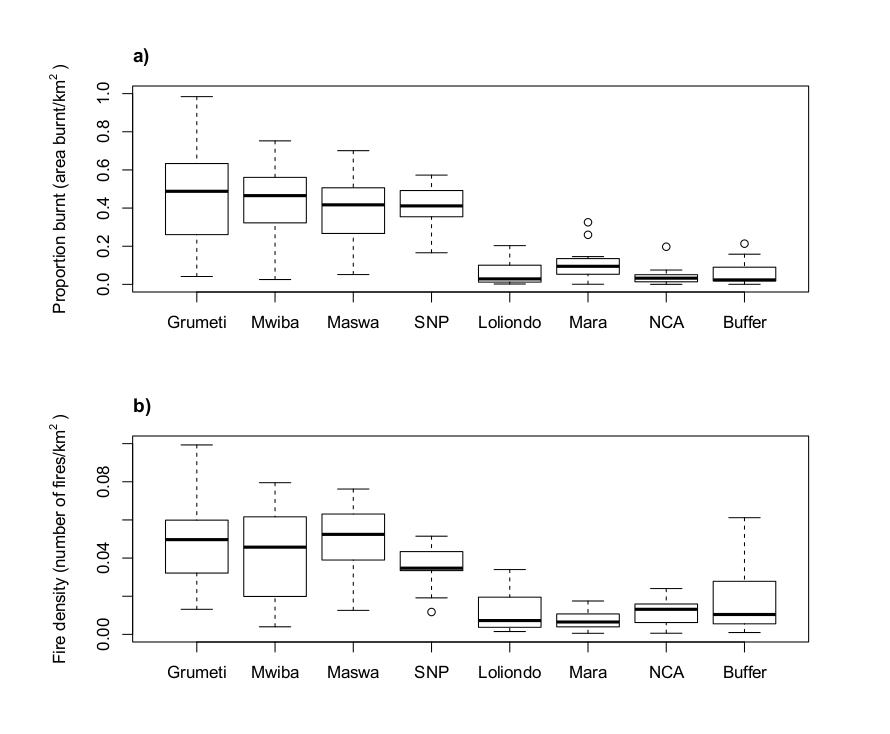

Supplement: Supplementary file 4 [file GCB-25-3406-s004.tiff]

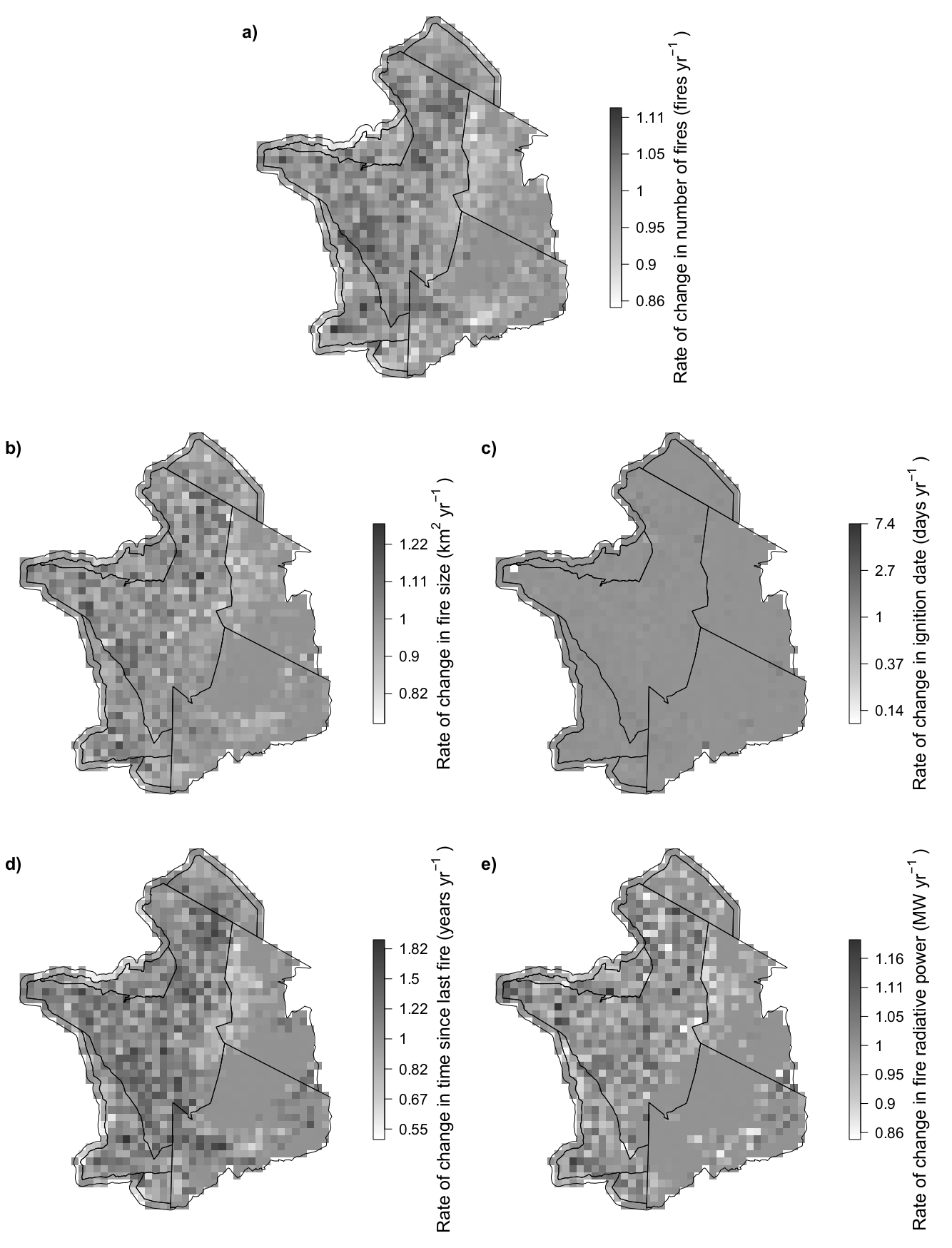

Supplement: Supplementary file 5 [file GCB-25-3406-s005.tiff]

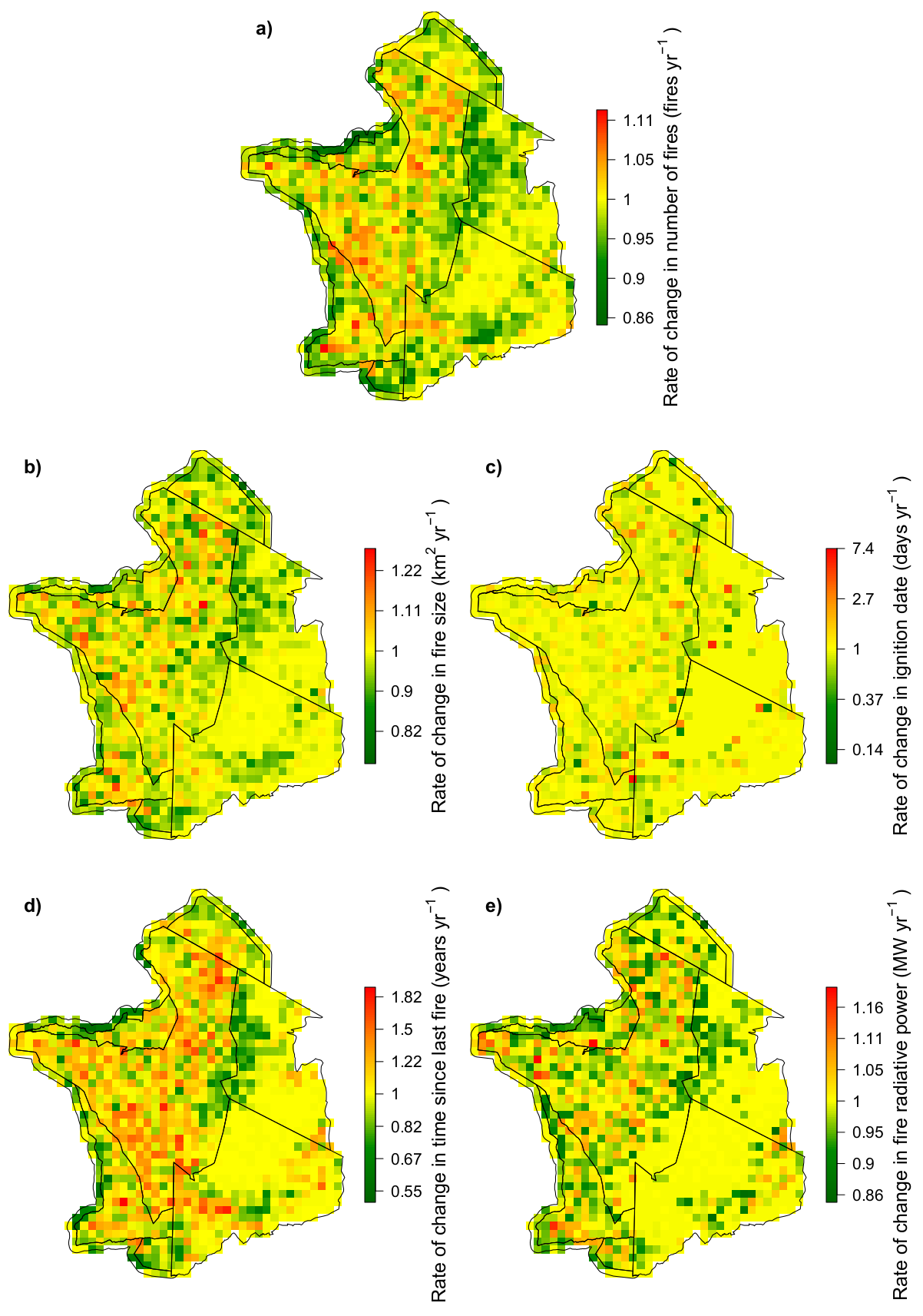

Supplement: Supplementary file 6 [file GCB-25-3406-s006.tiff]

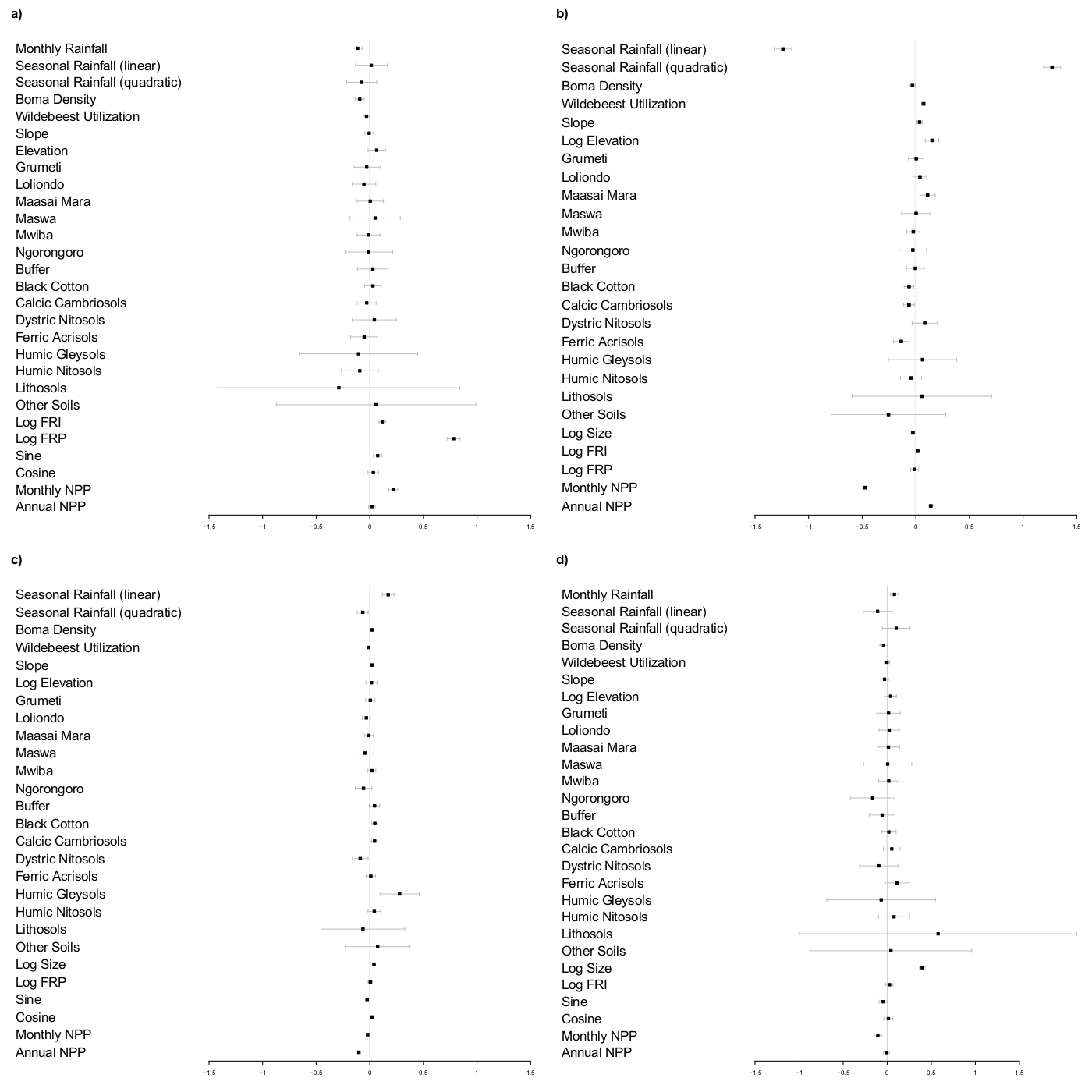

Supplement: Supplementary file 7 [file GCB-25-3406-s007.tiff]
