# Supplementary material for: Anthropogenic modifications to fire regimes in the wider Serengeti‐Mara ecosystem
Source: Glob Chang Biol. 2019 Jul 8;25(10):3406–23. doi: 10.1111/gcb.14711 (PMC6852266; doi:10.1111/gcb.14711)
Supplement: Supplementary file 8 [file GCB-25-3406-s008.pdf]

SUPPLEMENTARY MATERIAL

ADDITIONAL FIGURES AND TABLES

Table 1: The error structures, link functions and data transformations for all GLMs.

	Model	Error structure	Link function	Data transformation
Preliminary models	Change in fire size	Gaussian	Identity	Log
Preliminary models	Change in time since last fire	Gaussian	Identity	Log
Preliminary models	Change in radiative power	Gaussian	Identity	Log
Preliminary models	Change in number of fires	Gaussian	Identity	Log
Preliminary models	Change in area burnt	Gamma	Log	-
Preliminary models	Change in boma density	Poisson	Log	-
Preliminary models	Change in mean annual rainfall	Gaussian	Identity	-
	Final Model	Gaussian	Identity	-

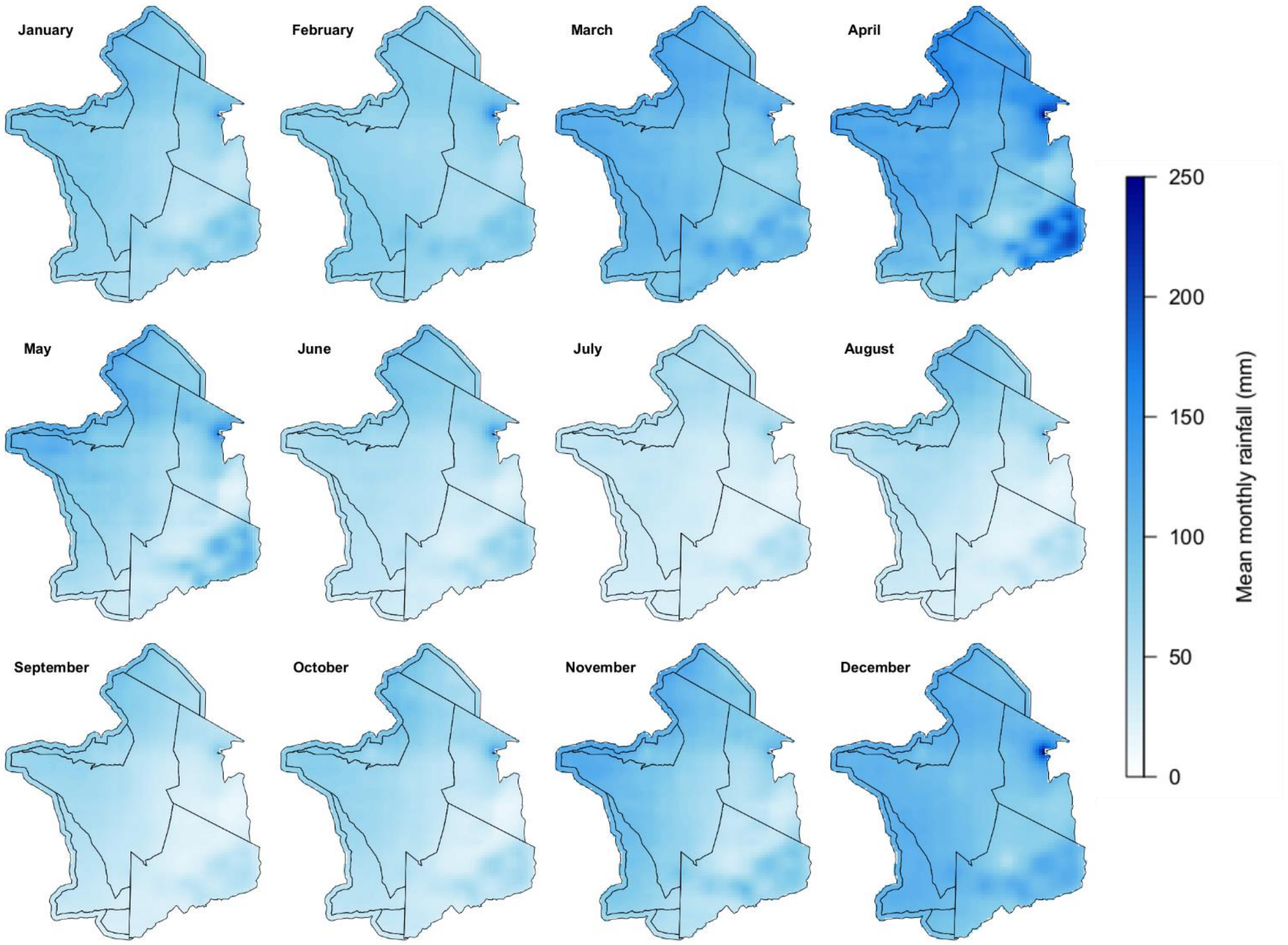

Figure 1: The mean monthly rainfall throughout our study period.

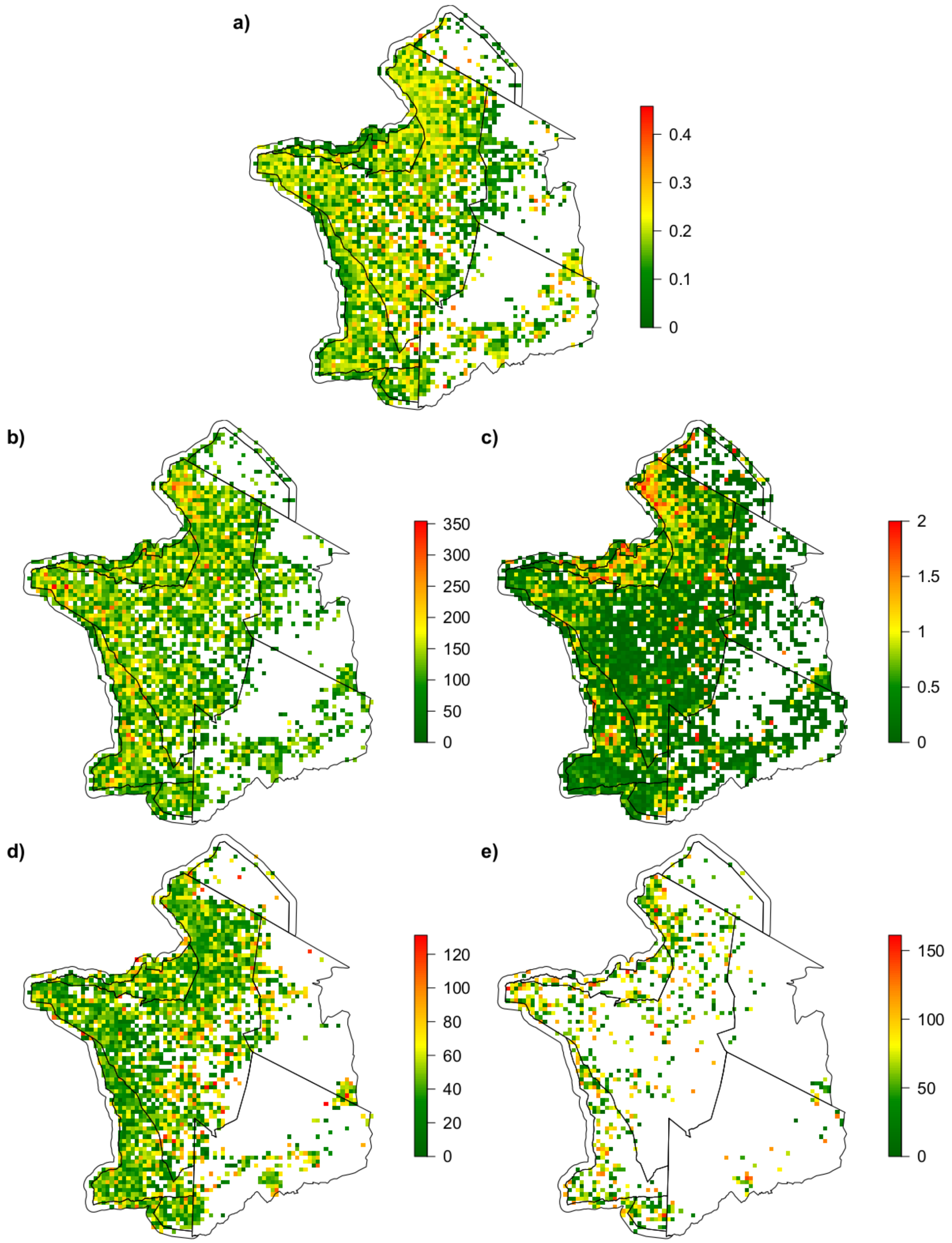

Figure 2: Variability in: a) the number of fires, b) fire size, c) ignition date, d) time since last fire, and e) fire radiative power. Note ignition date is more variable in the north-west. Variability is measured by the coefficient of variation except for ignition date, for which the angular variance was used.

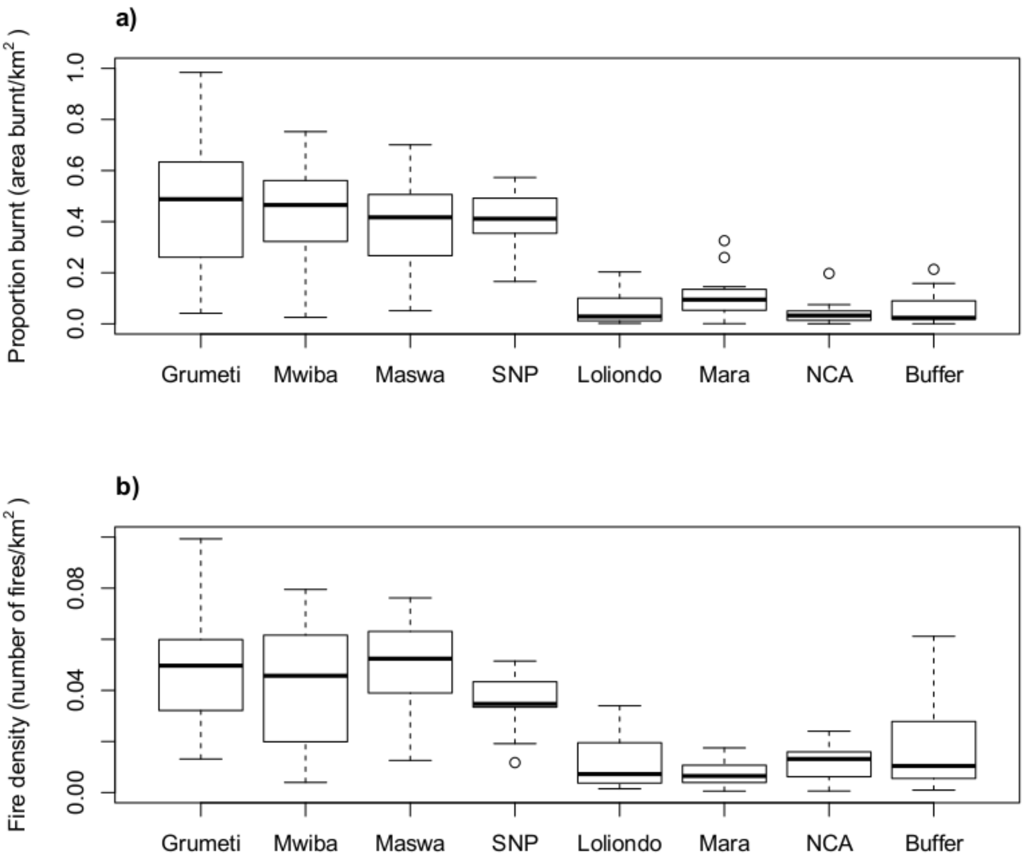

Figure 3: a) The proportion burnt and b) the number of fires per km^2 in each management unit during our study period.

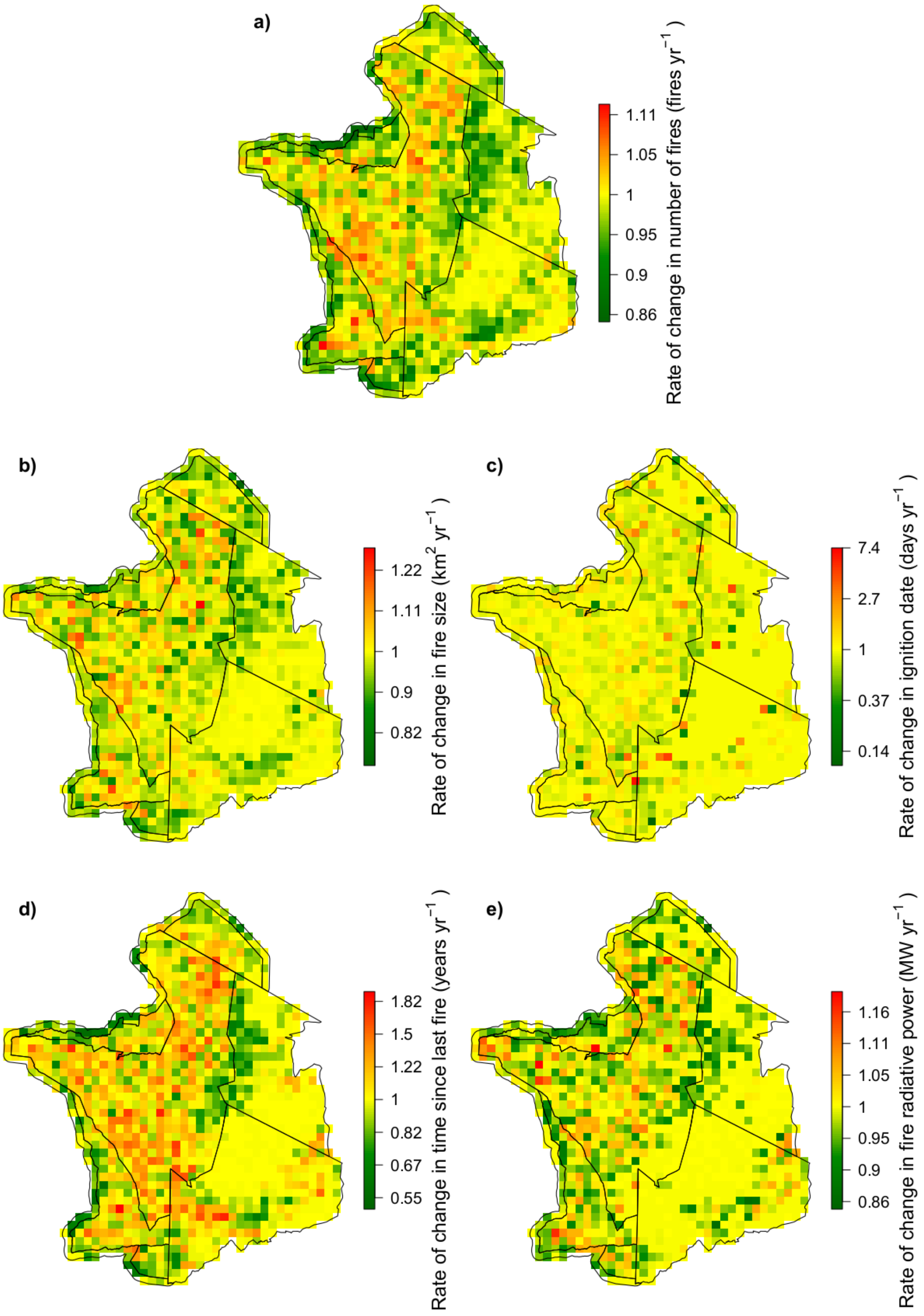

Figure 4: The rate of change in: a) the number of fires, b) median fire size, c) median ignition date, d) median time since last fire, and e) median fire radiative power.

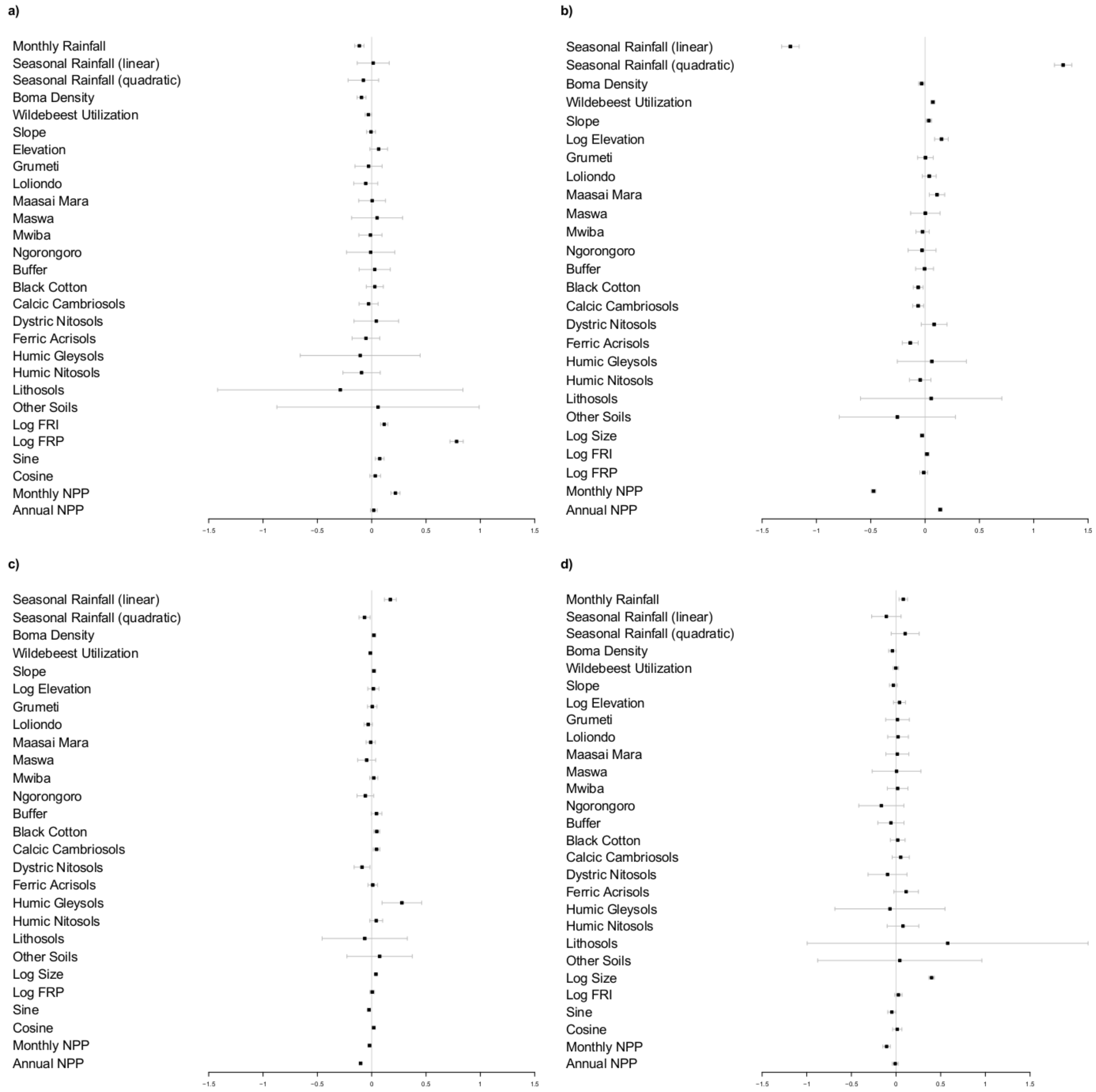

Figure 5: The 95% credible intervals for each fire trait from our spatial models: a) fire size, b) ignition date, c) time since last fire, and d) fire radiative power.

Table 2: The results of our GLM on the rate of change in the area burnt.

Variable	Df	Deviance	Residual Df	Residual deviance	Pr(>Chi)	Significance
NULL	-	-	1151	12.6030	-	-
Change in boma density	1	0.0268	1150	12.5762	0.007	**
Change in mean annual rainfall	1	0.0150	1149	12.5612	0.04	.
Management	7	0.9936	1142	11.5677	<0.001	***
Mean annual rainfall	1	0.0066	1141	11.5611	0.18	
Baseline area burnt	1	7.1431	1140	4.4180	<0.001	***
Change in boma density x mean annual rainfall	1	0.1071	1139	4.3109	<0.001	***
Change in boma density x baseline area burnt	1	0.0286	1138	4.2823	0.005	**
Change in mean annual rainfall x mean annual rainfall	1	0.0987	1137	4.1835	<0.001	***
